# Supplementary material for: A quantitative mass spectrometry-based approach to monitor the dynamics of endogenous chromatin-associated protein complexes
Source: Nat Commun. 2018 Jun 13;9:2311. doi: 10.1038/s41467-018-04619-5 (PMC5998130; doi:10.1038/s41467-018-04619-5)
Supplement: Supplementary file 2 — Supplementary Information [file 41467_2018_4619_MOESM2_ESM.pdf]

Supplementary Information for:

A quantitative mass spectrometry-based approach to monitor the dynamics of endogenous chromatin-associated protein complexes

Papachristou et al.

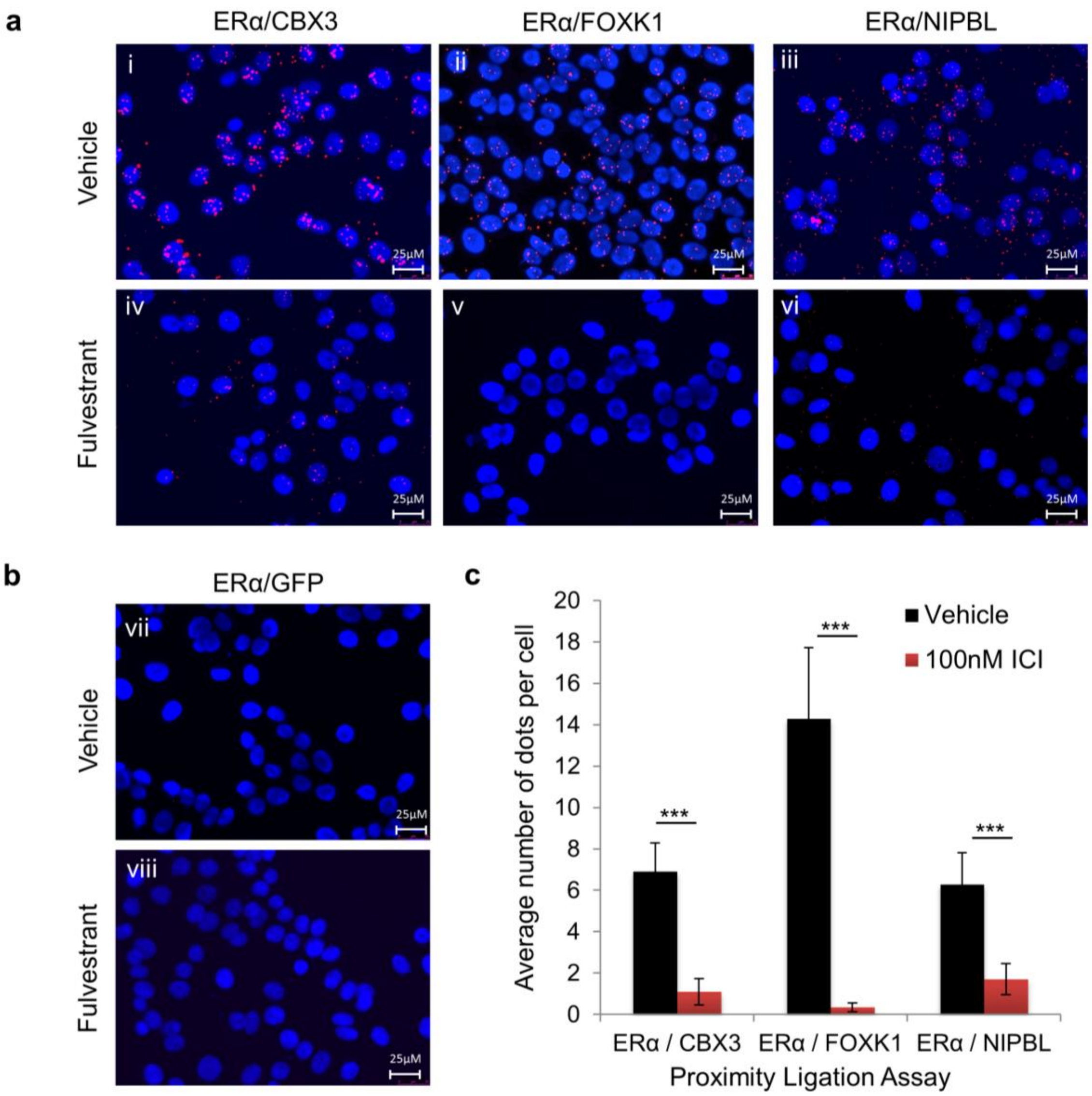

<u>Supplementary Figure 1.</u> Validation of novel ERα-associated cofactors using Proximity Ligation Assay (PLA). a) MCF7 cells were treated for three hours with vehicle (ethanol) (panels i–iii) or with 100nM Fulvestrant (ICI) (panels iv–vi) and were analysed by PLA to validate protein interactions between ERα and CBX3 (panels i and iv), FOXK1 (panels ii and v) and NIPBL (panels iii and vi). Images were acquired at 400x magnification. The experiment was performed in duplicate and the graphs are representative of one of the experiments. **b)** PLA assay using antibodies against ERα and GFP was used as a negative control (panels vii and viii). **c)** Quantification of the number of red PLA dots per cell was performed using Image J software (*** Student's t-test p-value < 0.001). The error bars indicate standard deviation (SD).

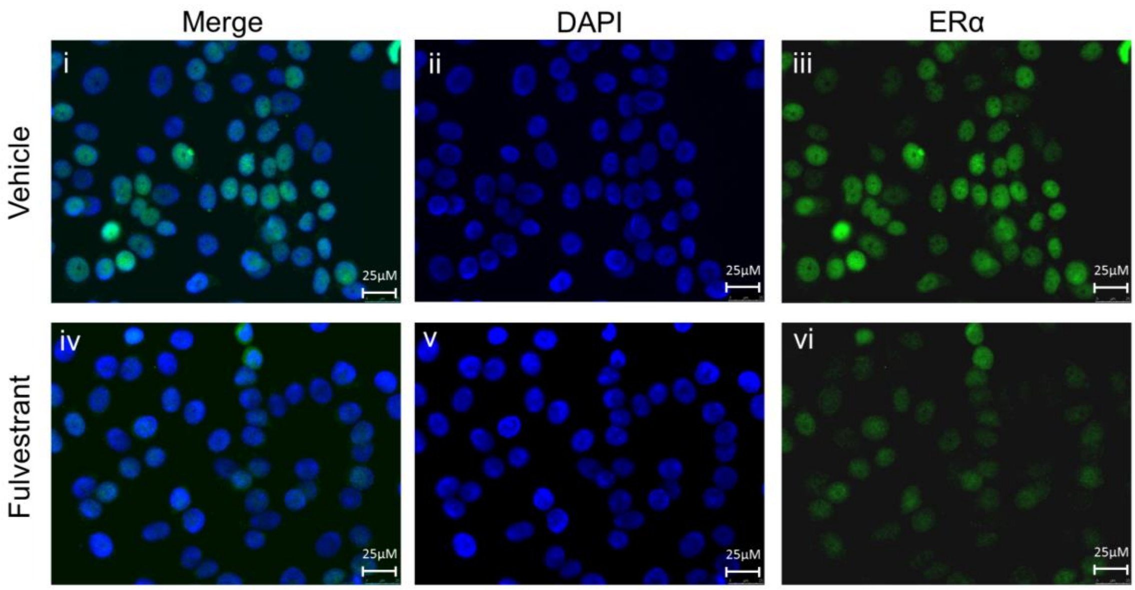

<u>Supplementary Figure 2.</u> Decrease in ERα protein levels upon Fulvestrant treatment. Immunofluorescence staining for ERα in MCF7 cells treated with vehicle (ethanol) (panels i–iii) or with 100nM Fulvestrant (panels iv–vi) for three hours.

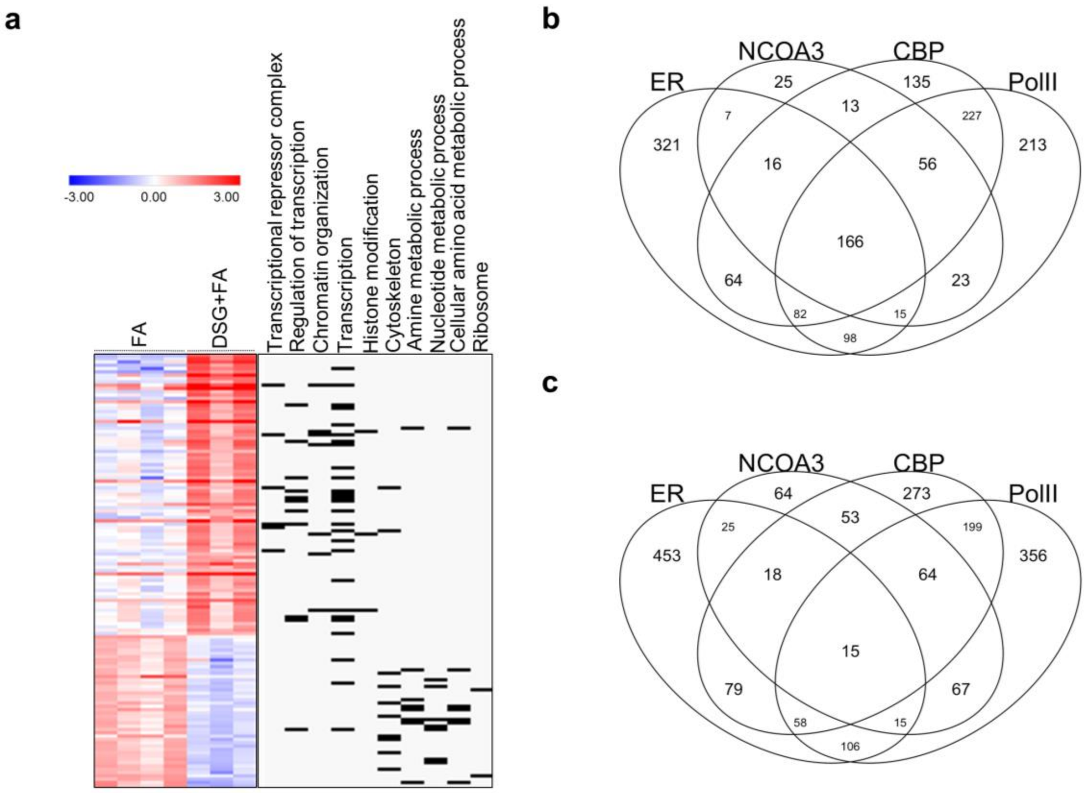

<u>Supplementary Figure 3.</u> Efficiency of double crosslinking and comparison of the four bait proteins. a) Comparison of double versus single crosslinking using qPLEX-RIME analysis showed an enrichment of proteins related to transcription regulation and chromatin organization. The scale bar represents $\log_2$-scaled protein abundance values. **b)** The venn diagram illustrates the comparison of the enriched interactors ($\log_2$Fold-Change>1, adj.p-value<0.05, > 1 unique peptide) of the four bait proteins (ERα, CBP, NCOA3, POLR2A). **c)** The venn diagram highlights the overlap of the four bait proteins using random selection of proteins identified in the four qPLEX-RIME experiments.

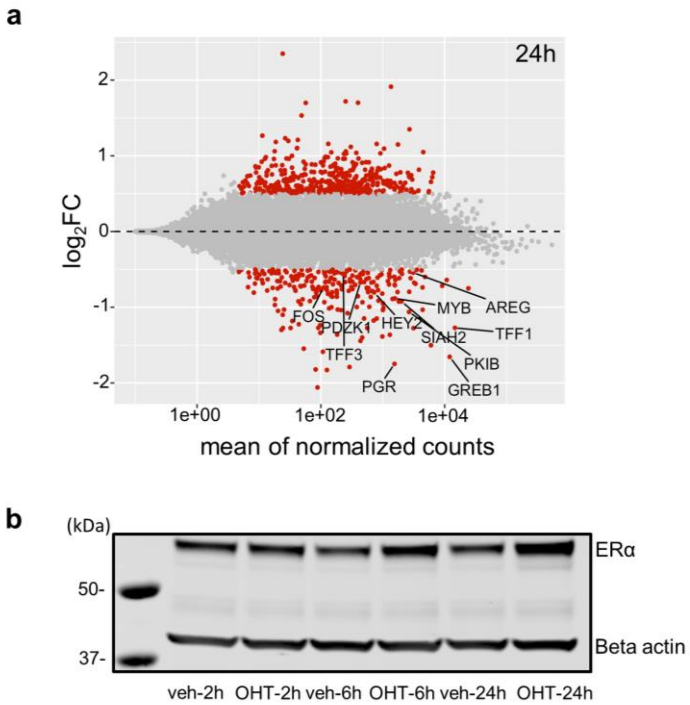

<u>Supplementary Figure 4.</u> RNA-seq analysis and profile of ERα total protein levels. a) Scatter plot summarizing the RNA-seq quantification results. Regulated genes are highlighted in red (|$\log_2$Fold-Change|>0.5, adj. p-value<0.05) and known ERα target genes are labelled. **b)** Western blot showing the temporal changes of ERα upon OHT treatment in whole cell lysate (MCF7).

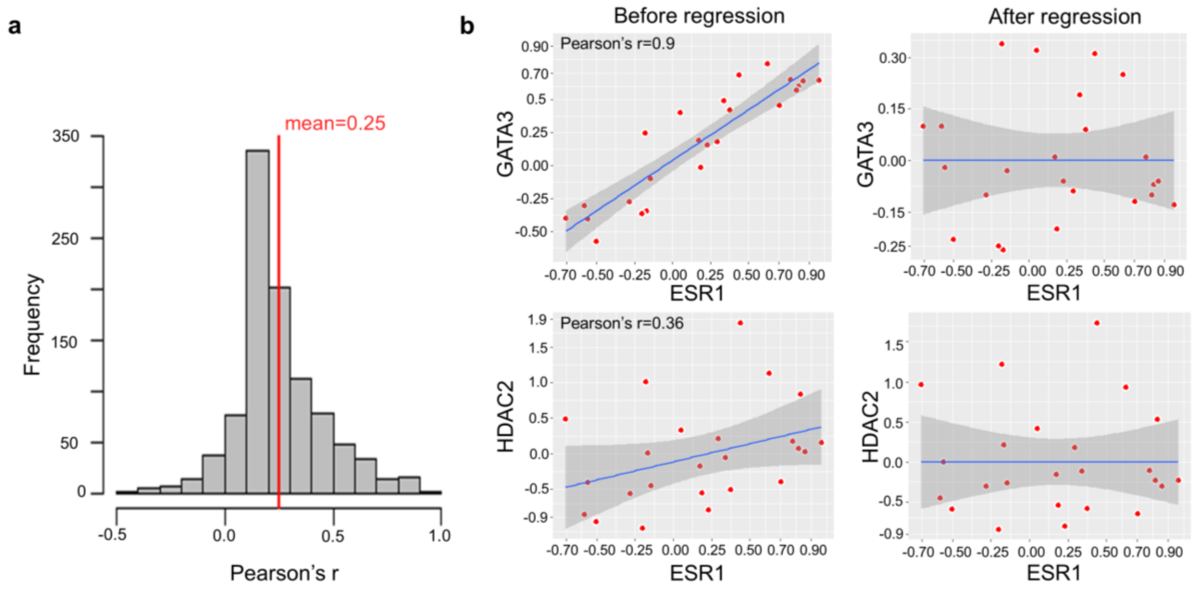

<u>Supplementary Figure 5.</u> Correction of quantitative results for the dependency on ERα target protein by linear regression. **a)** Histogram of Pearson's correlation coefficients between ERα protein profile and all ERα-enriched proteins. **b)** Scatter plots of ERα profile versus the raw (left panel) and corrected (right panel) quantitative values of GATA3 (with strong dependency on ERα) and HDAC2 (with low dependency on ERα).

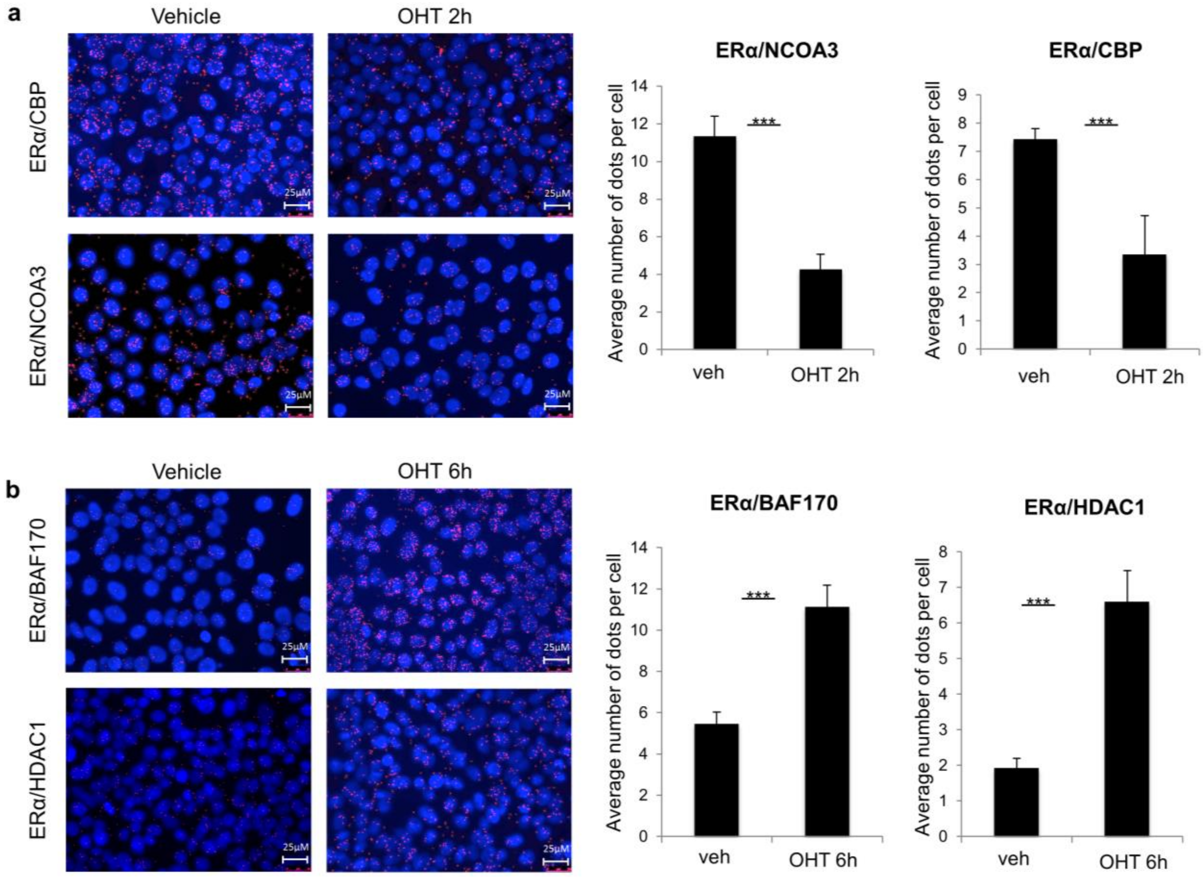

<u>Supplementary Figure 6.</u> Validation of temporal changes in the ERα complex upon OHT treatment. a) PLA assay showing the loss of NCOA3 and CBP from the ERα complex at 2h treatment with OHT compared to the respective control vehicle treatments (left panel). The experiment was performed in duplicate, and the graphs are representative of one of the experiments. Bar plots showing the quantification of the number of red PLA dots per cell normalized to ERα counts (single recognition PLA assay) using Image J software (***Student's t-test p-value < 0.001) (right panel). The error bars indicate standard deviation (SD). **b)** PLA assay showing the enrichment of BAF170 and HDAC1 from the ERα complex at 6h treatment with OHT compared to the respective control vehicle treatments (left panel). The experiment was performed in duplicate and the graphs are representative of one of the experiments. Bar plots showing the quantification of the number of red PLA dots per cell normalized to ERα counts (single recognition PLA assay) using Image J software (***Student's t-test p-value < 0.001) (right panel). The error bars indicate standard deviation (SD).

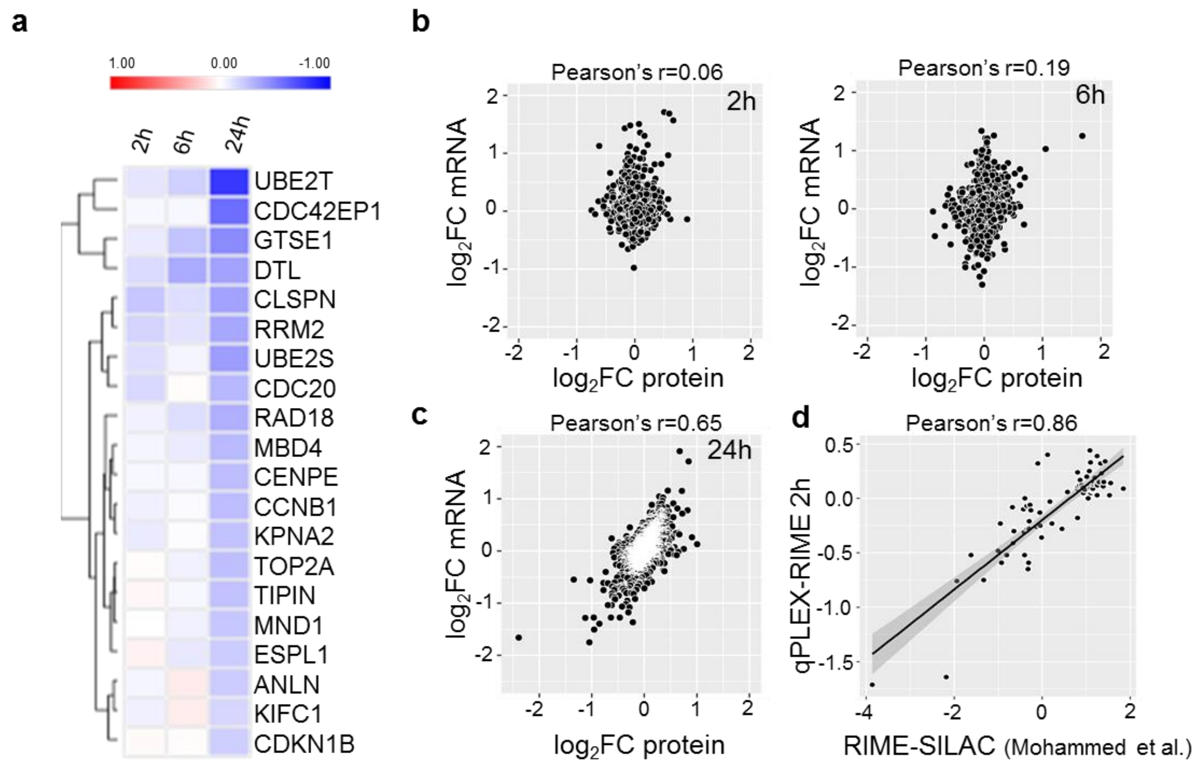

<u>Supplementary Figure 7.</u> Differentially regulated proteins and correlation with gene expression. a) Hierarchical clustering of down-regulated proteins involved in cell cycle at 24h using total proteome measurements. The scale bars represents log_2 ratios versus the vehicle treatment. **b)** Scatter plots of mRNA versus total protein at 2h and 6h treatment with OHT. **c)** Scatter plot of mRNA versus total protein after 24h treatment with OHT. **d)** Comparison of the time course qPLEX-RIME data at 2h OHT treatment with published RIME-SILAC data where MCF7 cells were treated with OHT for 3h showed a good correlation between the two datasets.

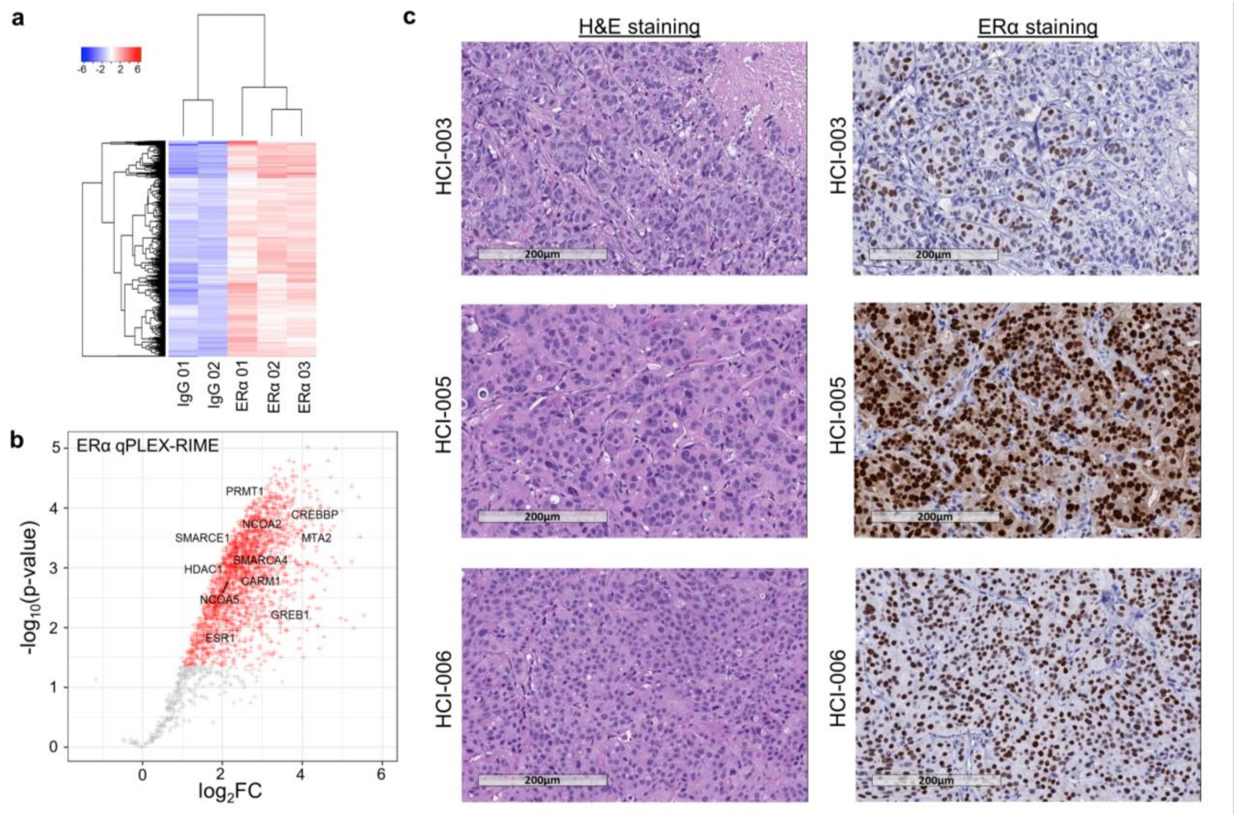

Supplementary Figure 8. ERα qPLEX-RIME application in PDX tissues. a) Hierarchical clustering of the qPLEX-RIME quantified ERα-enriched proteins in PDX tissues. The scale bar represents row-mean scaled log_2 values. **b)** Volcano plot summarising the quantitative results of qPLEX-RIME application in PDX tissues. Statistically significant proteins (log_2Fold-Change>1, adj. p-value<0.05) are shown in red color and several well-known ERα interactors are labelled. **c)** H&E (Haemotoxylin and Eosin) staining and ERα staining for the three independent ER-positive human Patient Derived Xenograft (PDX) tumours (HCI-003, HCI-005, HCI-006) that were used in the qPLEX-RIME experiment (magnification=20x, scale bar=200µm).

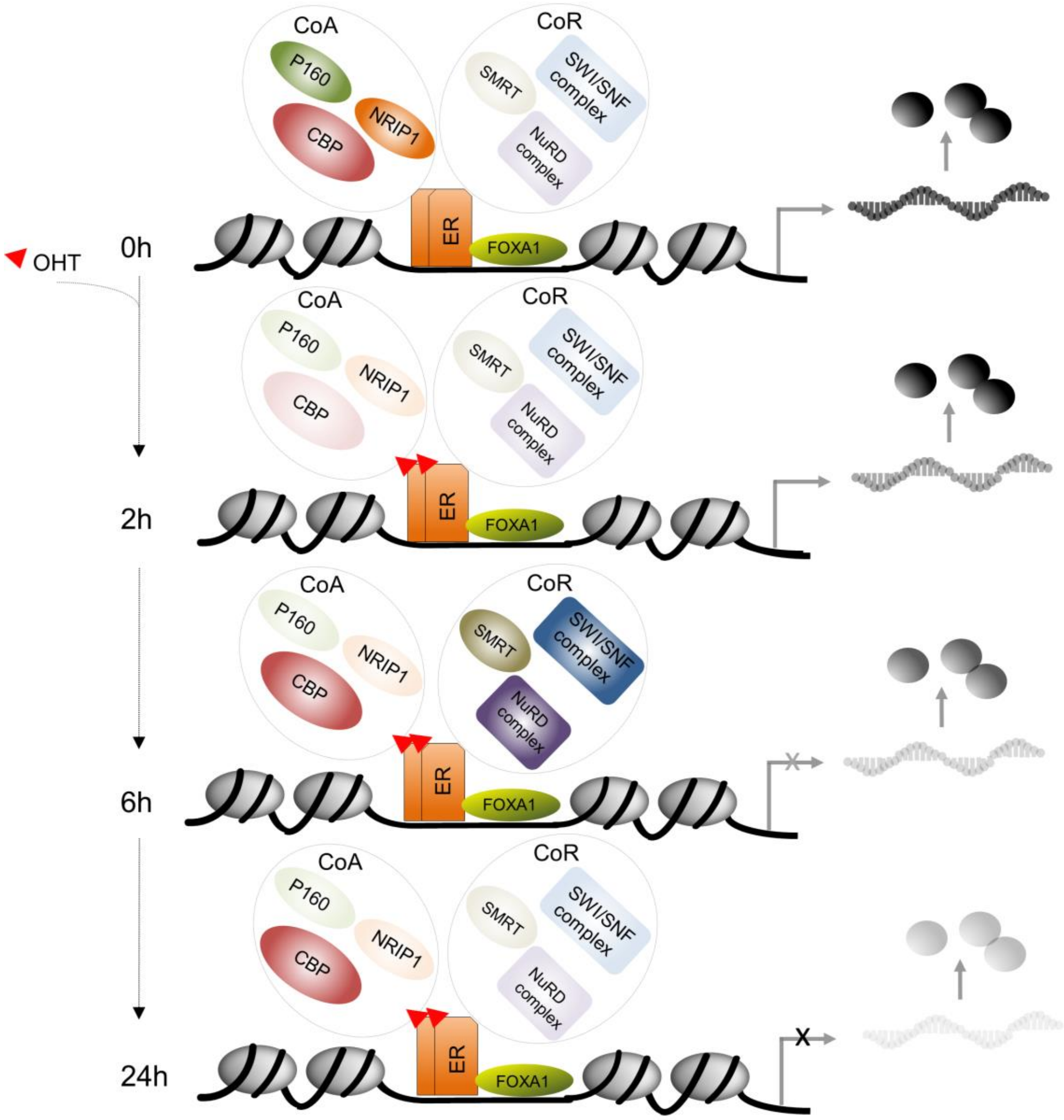

<u>Supplementary Figure 9.</u> A proposed model of 4-hydroxytamoxifen (OHT) mechanism. In untreated conditions (0h), both co-activators (CoA) and co-repressors (CoR) are interacting with ERα in the chromatin. The pioneer factor FOXA1 is on chromatin as well. After 2h treatment, a loss of co-activators such as NCOA3 and CBP takes place. At 6h, an enhanced recruitment of the NuRD and SWI/SNF complexes is observed coinciding with the enrichment of the NCOR2 corepressor. The proteins NCOA3 and NRIP1 remain at low levels in the interactome. At 24h, we observed a full restoration of the ERα complex with the exception of the NCOA3 and NRIP1 proteins, which were maintained at decreased amounts in the complex. After 24h treatment, a downregulation of ERα target genes at transcript and protein level is observed. In the cartoon, intense and faint colours indicate enrichment or loss respectively.

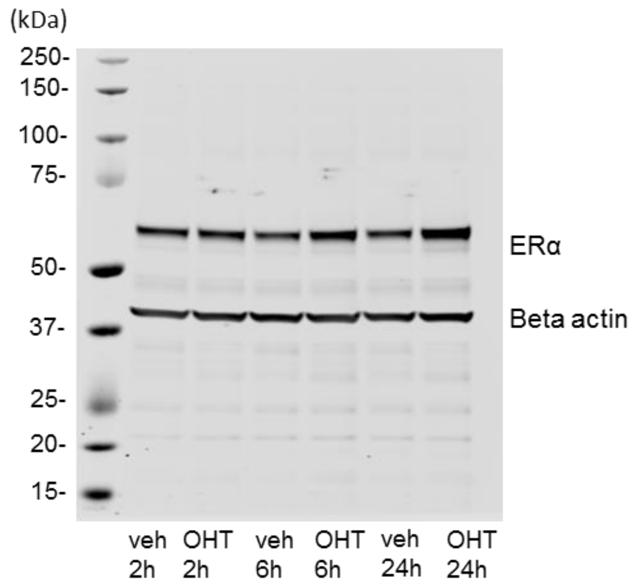

<u>Supplementary Figure 10.</u> ERα total protein levels upon OHT treatment. Uncropped scan of the western blot, showing the temporal changes of ERα upon OHT treatment in whole cell lysate (MCF7).

Supplementary Note 1: qPLEXanalyzer

Contents

1 **Overview** 1

2 **Import quantitative dataset** 2

3 **Quality control** 2

4 **Data normalization** 8

5 **Aggregation of peptide intensities into protein intensities** 11

6 **Regression Analysis** 12

7 **Differential statistical analysis** 13

1 Overview

This document provides brief tutorial of the *qPLEXanalyzer* package, a toolkit with multiple functionalities, for statistical analysis of qPLEX-RIME proteomics data (see ?qPLEXanalyzer at the R prompt for a brief overview). The qPLEX-RIME approach combines the RIME method with multiplex TMT chemical isobaric labelling to study the dynamics of chromatin-associated protein complexes. The package can also be used for isobaric labelling (TMT or iTRAQ) based total proteome analysis.

- Import quantitative dataset: A pre-processed quantitative dataset generated from MaxQuant, Proteome Discoverer or any other proteomic software consisting of peptide intensities with associated features along with sample meta-data information can be imported by *qPLEXanalyzer*.
- Quality control: Computes and displays quality control statistics plots of the quantitative dataset.
- Data normalization: Quantile normalization, central tendencies scaling and linear regression based normalization.
- Aggregation of peptide intensities into protein intensities
- Differential statistical analysis: *limma* based analysis to identify differentially abundant proteins.

```
library(qPLEXanalyzer)
library(grid)
data(human_anno)
data(exp2_Xlink)
```

2 Import quantitative dataset

MSnbase package by Laurent Gatto provides methods to facilitate reproducible analysis of MS-based proteomics data. *MSnSet* class of *MSnbase* provides architecture for storing quantitative MS proteomics data and the experimental meta-data. In *qPLEXanalyzer*, we store pre-processed quantitative proteomics data within this standardized object. The `convertToMSnset` function creates an *MSnSet* object from the quantitative dataset of peptides/protein intensities. This dataset must consist (or derived) of peptides identified with high confidence in all the samples.

The default input dataset is the pre-processed peptide intensities from MaxQuant, Proteome Discoverer or any other proteomic software (see ?convertToMSnset at the R prompt for more details). Only peptides uniquely matching to a protein should be used as an input. However, the protein level quantification by the aggregation of the peptide TMT intensities can also be used as input. Peptides/Protein intensities with missing values in one or more samples can either be excluded or included in the *MSnSet* object. If the missing values are kept in the *MSnSet* object, these must be imputed either by user defined methods or by those provided in *MSnbase* package. The downstream functions of *qPLEXanalyzer* expects no missing values in the *MSnSet* object.

The example dataset shown below is from an ER qPLEX-RIME experiment in MCF7 cells that was performed to compare two different ways of cell crosslinking: DSG/formaldehyde (double) or with formaldehyde alone (single). It consists of four biological replicates for each condition along with two IgG samples pooled from replicates of each group.

```
MSnset_data <- convertToMSnset(exp2_Xlink$intensities,
                               metadata=exp2_Xlink$metadata,
                               indExpData=c(7:16), Sequences=2, Accessions=6)
```

3 Quality control

Once an *MSnSet* object has been created, various descriptive statistics methods can be used to check the quality of the dataset. The `intensityPlot` function generates a peptide intensity distribution plot that helps in identifying samples with outlier distributions. Figure 1 shows the distribution of the log-intensity of peptides/proteins for each sample. An outlier sample DSG.FA.rep01 can be identified from this plot. IgG control samples representing low background intensities will have shifted/distinct intensity distribution curve as compared to other samples and should not be treated as an outlier.

```
intensityPlot(MSnset_data, title = "Peptide intensity distribution")
```

The intensities can also be viewed in the form of boxplots by `intensityPlot`. Figure 2 shows the distribution of peptides intensities for each sample. `rliPlot` can be used to visualise unwanted variation in a data set. It is similar to the relative log expression plot developed for microarray analysis - see Gandolfo and Speed (2018). Rather than examining gene expression, the RLI plot (Figure 3) uses the MS intensities for each peptide or the summarised protein intensities.

```
intensityBoxplot(MSnset_data, title = "Peptide intensity distribution")
```

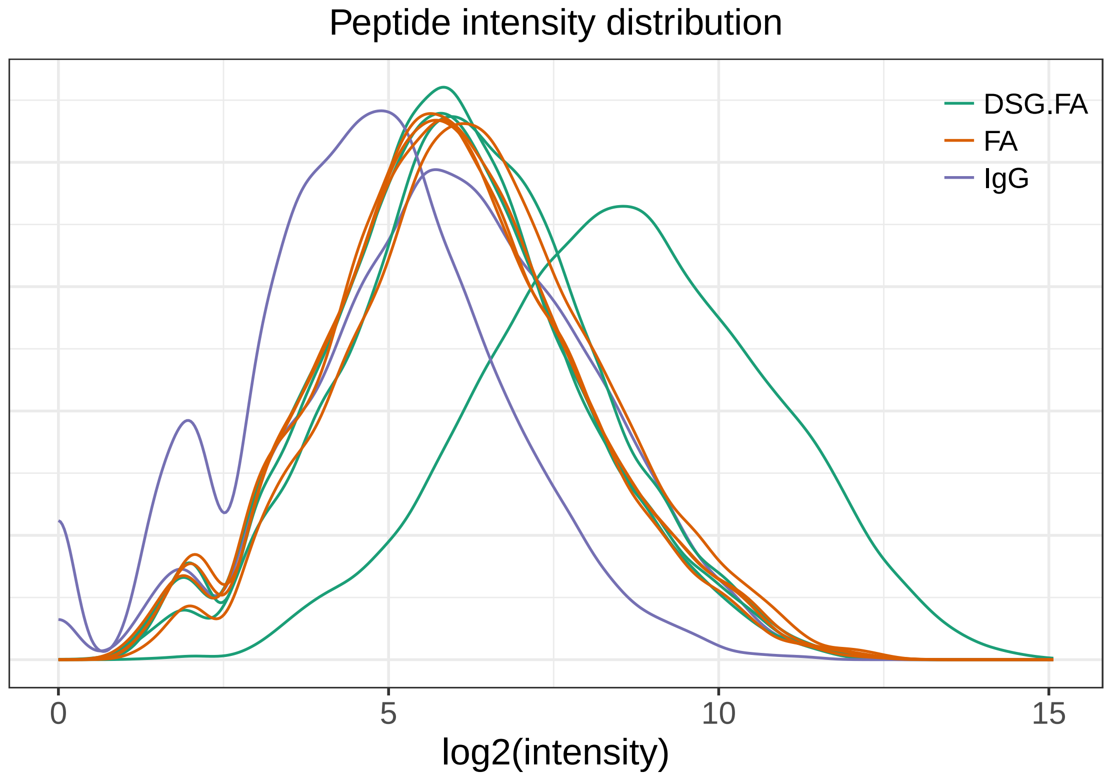

Figure 1: Density plots of raw intensities for TMT-10plex experiment.

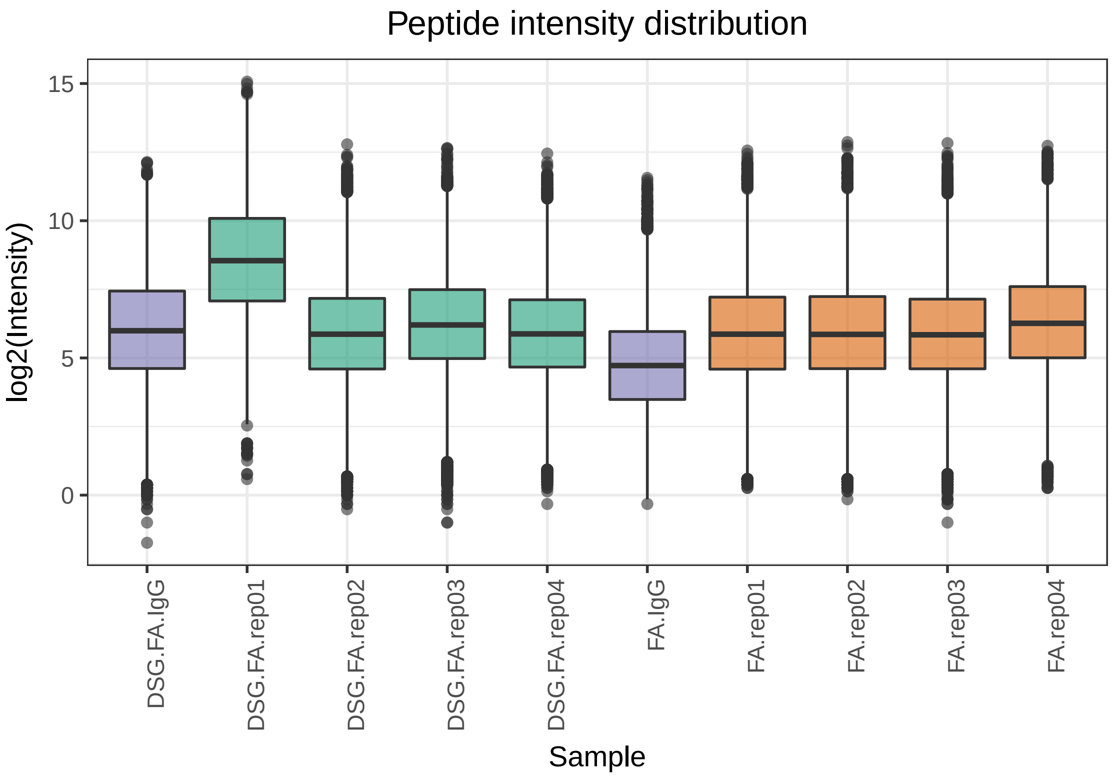

Figure 2: Boxplot of raw intensities for TMT-10plex experiment.

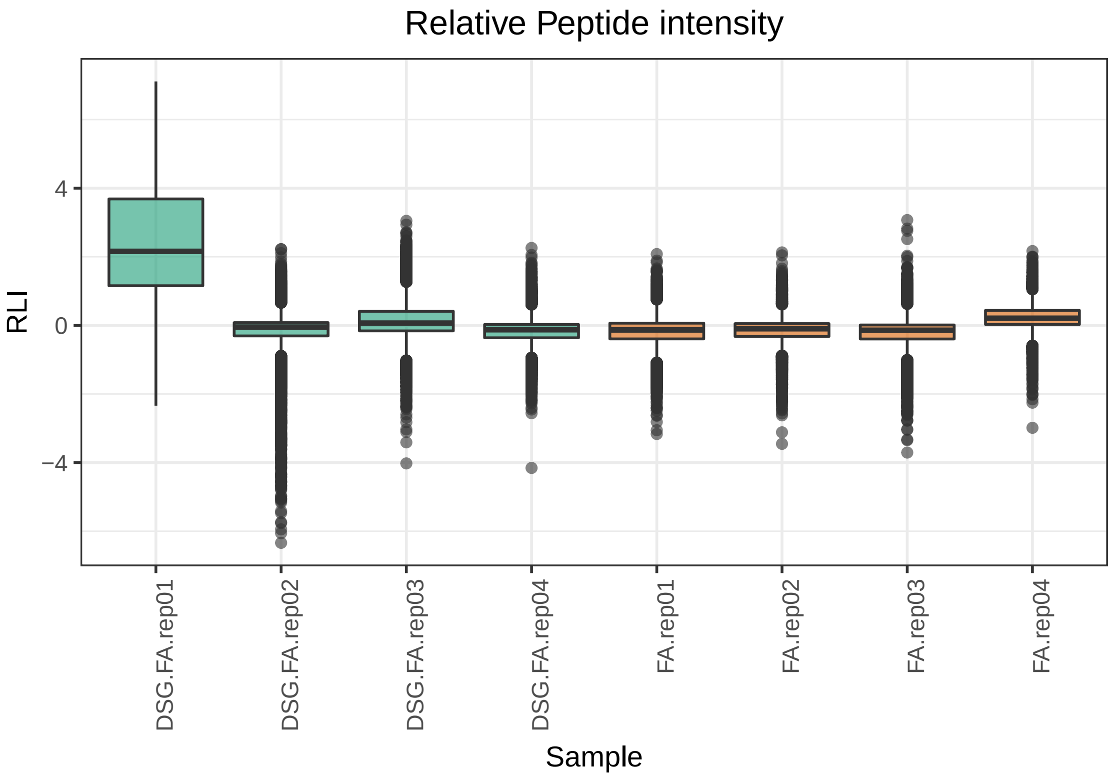

Figure 3: RLI of raw intensities for TMT-10plex experiment.

```
rliPlot(MSnset_data, title = "Relative Peptide intensity")
```

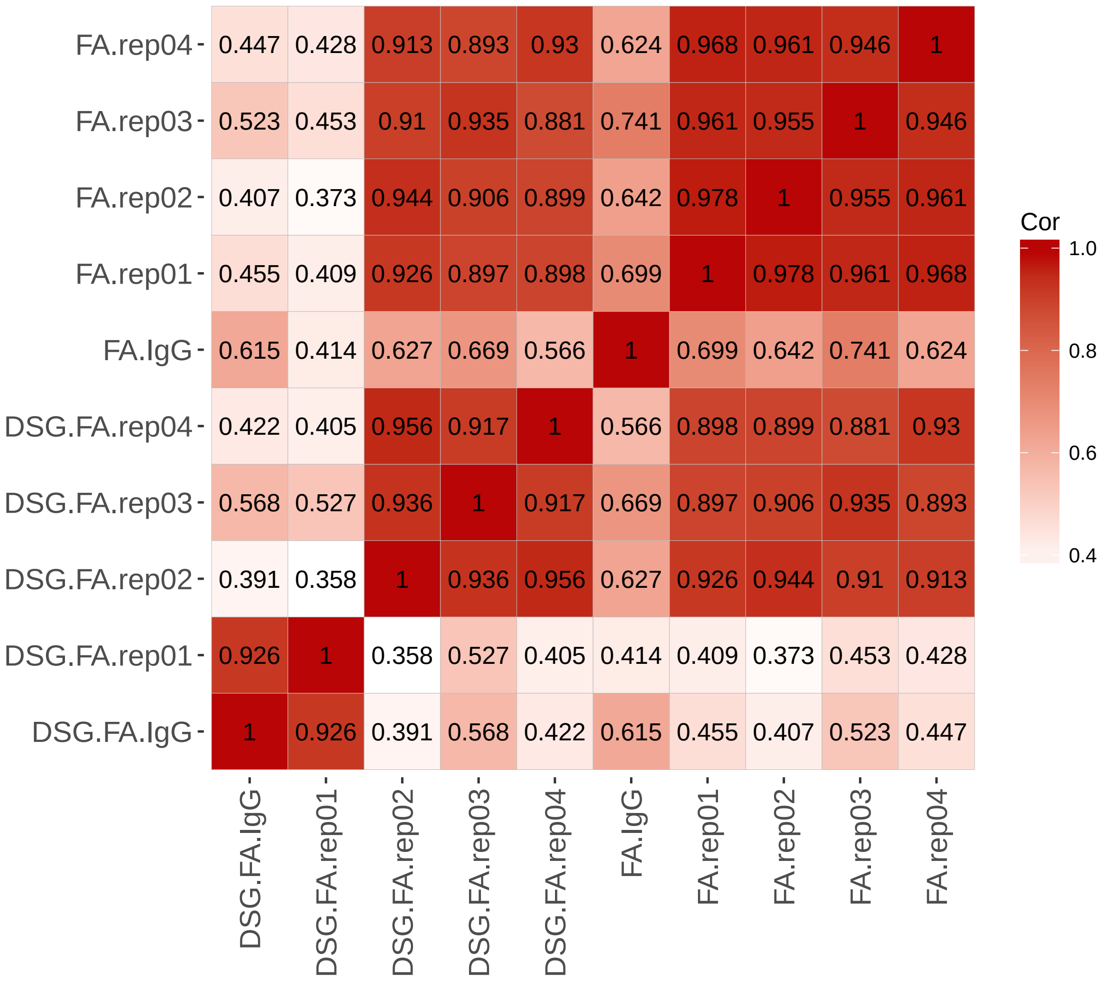

Figure 4: Correlation plot of peptide intensitites

A Correlation plot can be generated by `corrPlot` to visualize the level of linear association of samples within and between groups. The plot in Figure 4 displays high correlation among samples within each group, however an outlier sample is also identified in one of the groups (DSG.FA).

```
corrPlot(MSnset_data)
```

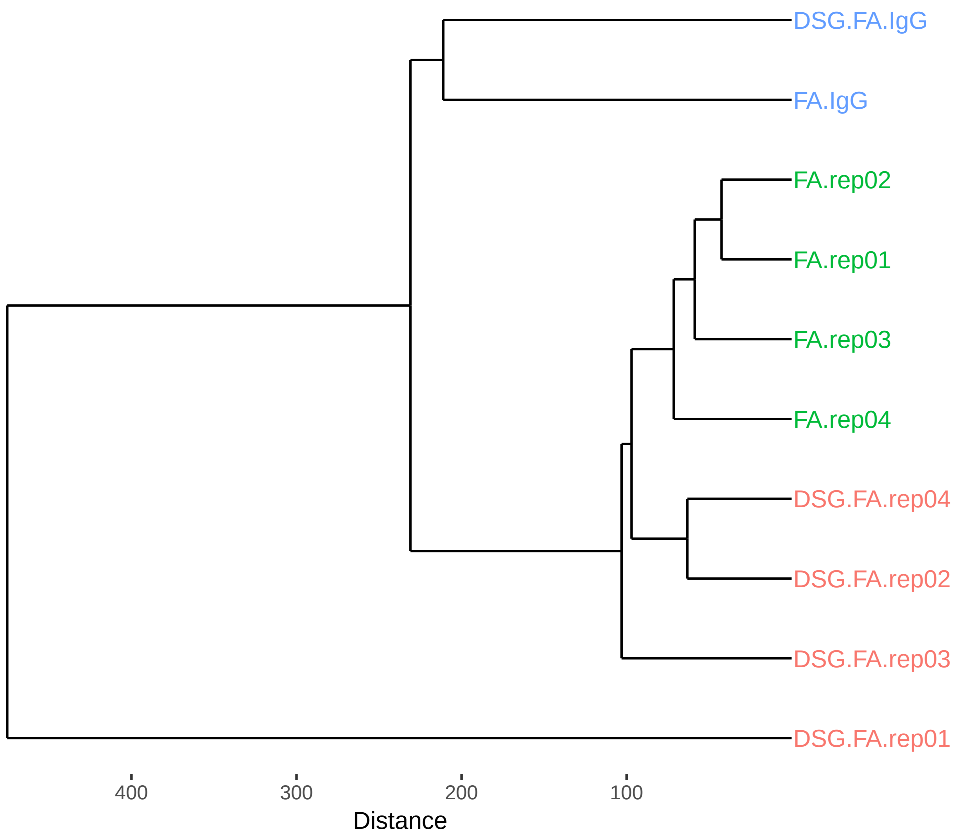

Figure 5: Clustering plot of peptide intensitites

Hierarchical clustering can be performed by `hierarchicalPlot` to produce a dendrogram displaying the hierarchical relationship among samples (Figure 5). The vertical axis shows the dissimilarity (measured by means of the Euclidean distance) between samples: similar samples appear on the same branches. Colors correspond to groups. To avoid error while generating hierarchical plot due to zero values in the dataset, a minimum value is added to the intensity data.

```
exprs(MSnset_data) <- exprs(MSnset_data)+0.01
suppressWarnings(hierarchicalPlot(MSnset_data))
```

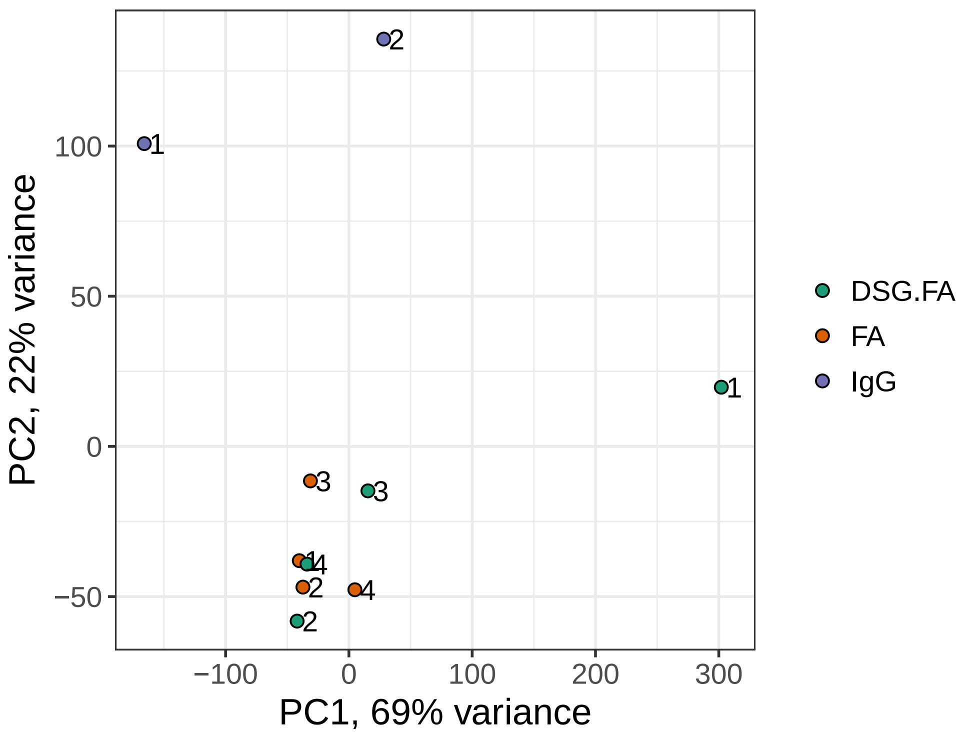

Figure 6: PCA plot of peptide intensitites

A visual representation of the scaled loading of the first two dimensions of a PCA analysis can be obtained by `pcaPlot` (Figure 6). Co-variances between samples are approximated by the inner product between samples. Highly correlated samples will appear close to each other. The samples could be labeled by name, replicate, group or experiment run allowing for identification of potential batch effects.

```
pcaPlot(MSnset_data, labelColumn="BioRep", pointsize=2)
```

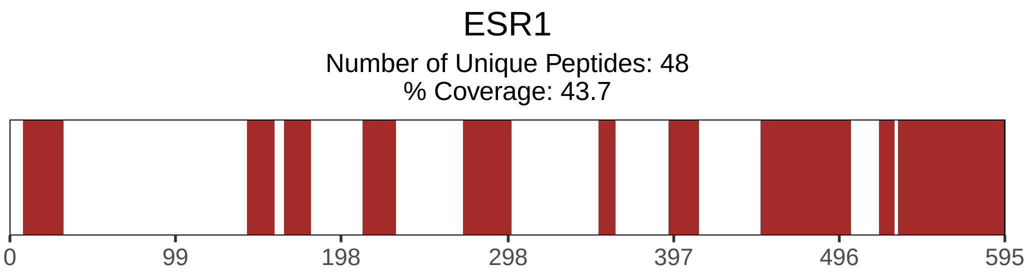

Figure 7: Peptide sequence coverage plot

To check the efficiency of the immunoprecipitation approach in the qPLEX-RIME method, a plot with the peptide sequence coverage of the bait protein can be illustrated. `coveragePlot` allows to plot peptide sequence coverage for the bait protein (Figure 7). The plot shows the location of peptides that have been identified with high confidence across the protein sequence and the corresponding percentage of the coverage. For a better evaluation of the pull down assay we can compare the observed bait protein coverage with the theoretical coverage.

```
mySequenceFile <- system.file('extdata', "P03372.fasta", package="qPLEXanalyzer")
coveragePlot(MSnset_data, ProteinID="P03372", ProteinName="ESR1",
             fastaFile=mySequenceFile)
```

4 Data normalization

The data can be normalized to remove experimental artifacts (e.g. differences in sample loading variability, systemic variation) in order to separate biological variations from those introduced during the experimental process. This would improve downstream statistical analysis to obtain more accurate comparisons. Different normalization methods can be used depending on the data.

- Quantiles: The peptide intensities are roughly replaced by the order statistics on their abundance. The key assumption underneath is that there are only few changes between different groups. This normalization technique has the effect of making the distributions of intensities from the different samples identical in terms of their statistical properties. It is the strongest normalization method and should be used carefully as it erases most of the difference between the samples. We would recommend using it only for total proteome but not for qPLEX-RIME data.
- Mean / median scaling: In this normalization method the central tendencies (mean or median) of the samples are aligned. The central tendency for each sample is computed and log transformed. A scaling factor is determined by subtracting from each central tendency the mean of all the central tendencies. The raw intensities are then divided by the scaling factor to get normalized ones.
- Row scaling: In this normalization method each peptide/protein intensity is divided by the mean/median of its intensity across all samples and log2 transformed.

It is imperative to check the intensity distribution plot and PCA plot before and after normalization to verify its effect on the dataset. In qPLEX-RIME data, the IgG (or control samples) should be normalized separately from the bait protein pull-down samples. As IgG samples represent the low background intensity, their intensity distribution profile is different from bait pull-downs. Hence, normalizing the

two together would result in over-correction of the IgG intensity resulting in inaccurate computation of differences among groups. However, if no normalization is necessary, skip this step and move to aggregation of peptides. For this dataset, an outlier sample was identified by quality control plots and removed from further analysis. Figure 8 displays the effect of various normalization methods on the peptide intensities distribution.

```
MSnset_data <- MSnset_data[,-5]
p1 <- intensityPlot(MSnset_data, title = "No normalization")
MSnset_norm_q <- normalizeQuantiles(MSnset_data)
p2 <- intensityPlot(MSnset_norm_q, title = "Quantile")
MSnset_norm_ns <- normalizeScaling(MSnset_data,func=median)
p3 <- intensityPlot(MSnset_norm_ns, title = "Scaling")
MSnset_norm_gs <- groupScaling(MSnset_data,func=median, Grp="SampleGroup")
p4 <- intensityPlot(MSnset_norm_gs, title = "WithinGrp Scaling")
grid.newpage()
pushViewport(viewport(layout = grid.layout(nrow = 2, ncol = 2)))
 define_region <- function(row, col){
   viewport(layout.pos.row = row, layout.pos.col = col)
}
print(p1, vp = define_region(row = 1, col = 1))
print(p2, vp = define_region(row = 1, col = 2))
print(p3, vp = define_region(row = 2, col = 1))
print(p4, vp = define_region(row = 2, col = 2))
```

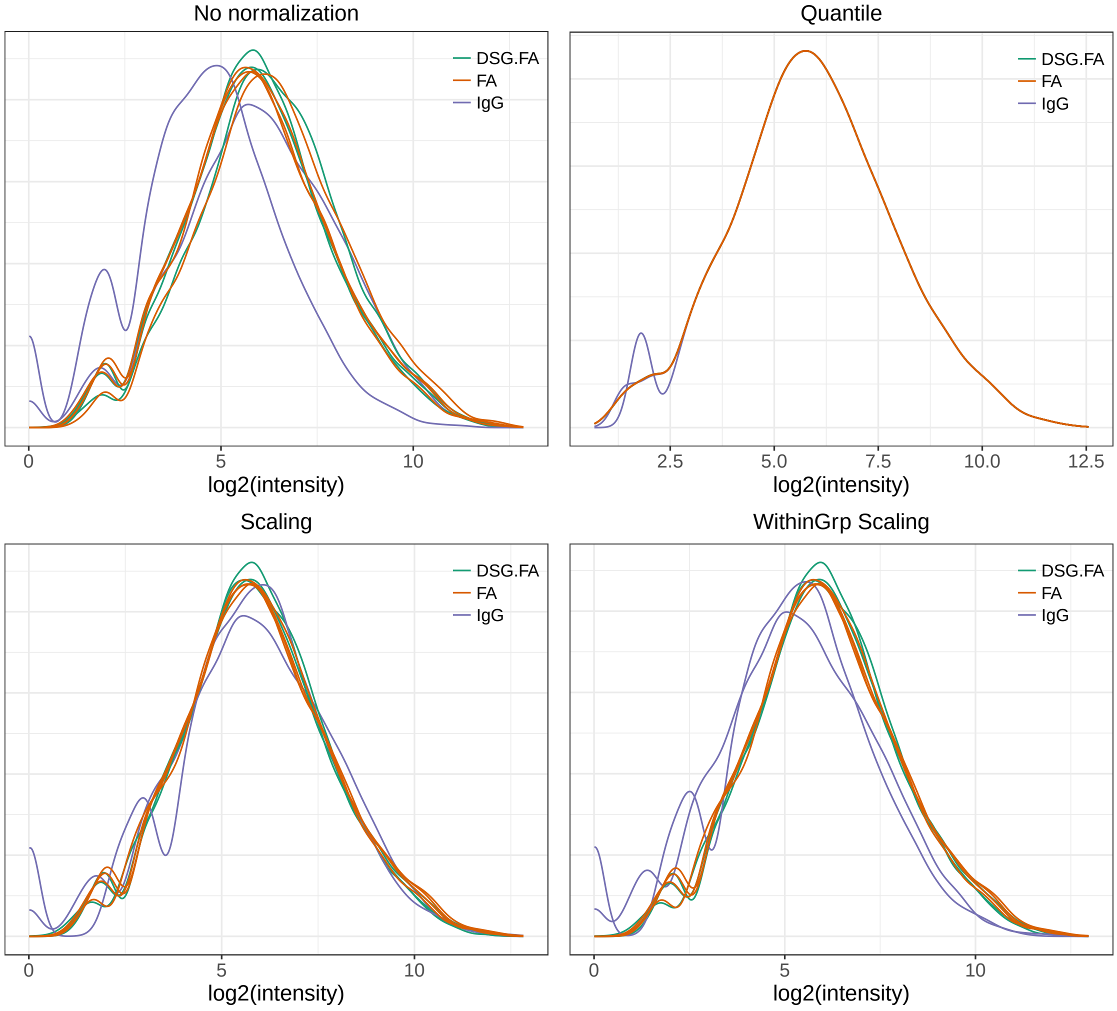

Figure 8: Peptide intensity distribution with various normalization methods

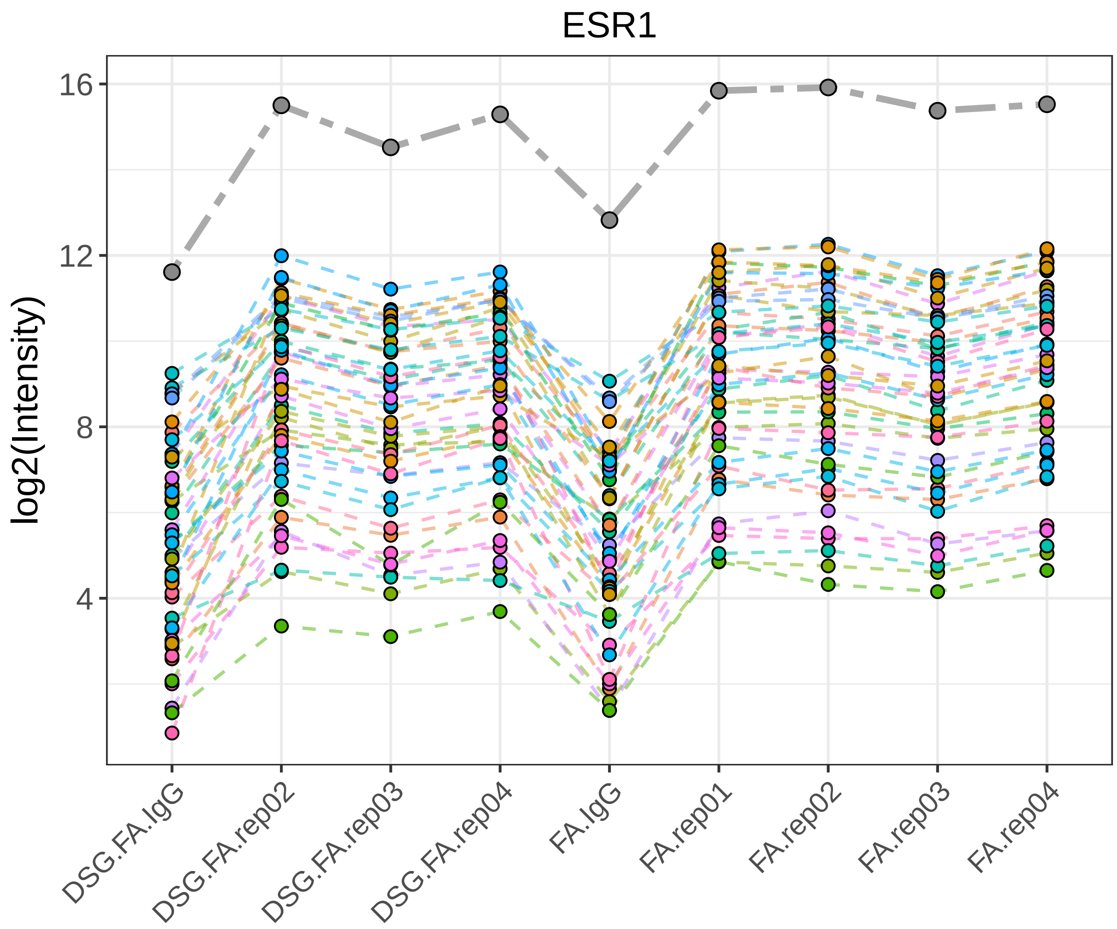

Figure 9: Summarized protein intensity

5 Aggregation of peptide intensities into protein intensities

The quantitative dataset could consist of peptide or protein intensities. If the dataset consists of peptide information, they can be aggregated to protein intensities for further analysis. For this, an annotation file consisting of proteins with unique ID must be provided. An example file can be found with the package corresponding to uniprot annotation of human and mouse proteins. It consists of four columns: "Protein", "Gene", "Description" and "GeneSymbol". The `summarizeIntensities` function expects an annotation file in this format. The aggregation can be performed by sum, mean or median of the raw or normalized peptide intensities. The summarized intensity for a selected protein could be visualized using `peptideIntensityPlot`. It plots all peptides intensities for a selected protein alongwith summarized intensity across all the samples (Figure 9).

```
MSnset_Pnorm <- summarizeIntensities(MSnset_norm_gs, sum, human_anno)
```

```
peptideIntensityPlot(MSnset_data,combinedIntensities=MSnset_Pnorm,
                                ProteinID="P03372", ProteinName= "ESR1")
```

6 Regression Analysis

To correct for the potential dependency of immunoprecipitated proteins (in qPLEX-RIME) on the bait protein, a linear regression method is available in *qPLEXanalyzer*. The `regressIntensity` function performs a regression analysis in which bait protein levels is the independent variable (x) and the profile of any other protein is the dependent variable (y). The residuals of the y=ax+b linear model represent the protein quantification profiles that are not driven by the amount of the bait protein.

The advantage of this approach is that proteins with strong dependency on the target protein are subjected to significant correction, whereas proteins with small dependency on the target protein are slightly corrected as opposed to the use of a standard correction factor, which would have the same effect to all proteins. The control samples (such as IgG) should be excluded from the regression analysis. The `regressIntensity` function also generates the plot displaying the correlation between bait and other protein before and after applying this method (Figure 10).

The example dataset shown below is from ER qPLEX-RIME experiments in MCF7 cells to investigate the dynamics of the ER complex assembly upon 4-hydroxytamoxifen (OHT) treatment at 2h, 6h and 24h or at 24h post-treatment with the vehicle alone (ethanol). It consists of six biological replicates for each condition spanned over three TMT experimental along with two IgG mock pull down samples in each experiment.

```
data(exp3_OHT_ESR1)
MSnset_reg <- convertToMSnset(exp3_OHT_ESR1$intensities_qPLEX2,
                              metadata=exp3_OHT_ESR1$metadata_qPLEX2,
                              indExpData=c(7:16), Sequences=2, Accessions=6)
MSnset_P <- summarizeIntensities(MSnset_reg, sum, human_anno)
MSnset_P <- rowScaling(MSnset_P, mean)
IgG_ind <- which(pData(MSnset_P)$SampleGroup == "IgG")
Reg_data <- regressIntensity(MSnset_P, controlInd=IgG_ind, ProteinId="P03372")
```

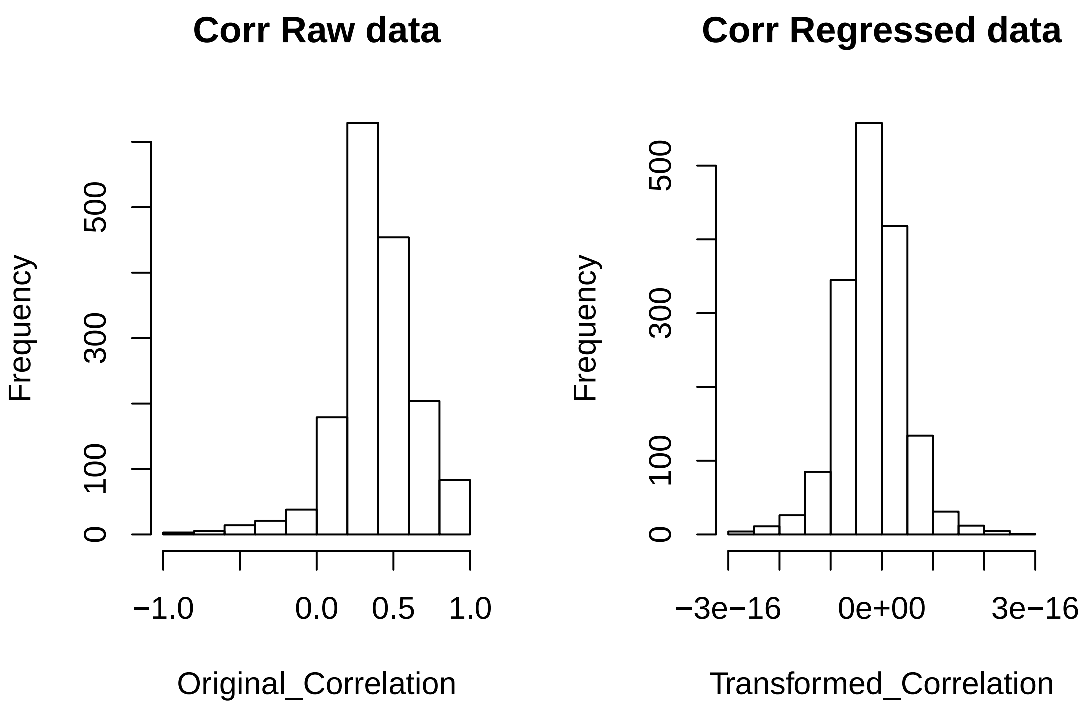

Figure 10: Correlation between bait protein and enriched proteins before and after regression

7 Differential statistical analysis

A statistical analysis for the identification of differentially regulated or bound proteins is carried out using *limma* based analysis. It uses linear models to assess differential expression in the context of multifactor designed experiments. Firstly, a linear model is fitted for each protein where the model includes variables for each group and MS run. Then, log2 fold changes between comparisons are estimated using `computeDiffStats`. Multiple testing correction of p-values are applied using the Benjamini-Hochberg method to control the false discovery rate (FDR). Finally, `getContrastResults` is used to get contrast specific results.

The qPLEX-RIME experiment can consist of IgG mock samples to discriminate non-specific binding. The controlGroup argument within `getContrastResults` function allows you to specify this group (such as IgG). It then uses the mean intensities from the fitted linear model to compute log2 fold change between IgG and each of the groups. The maximum log2 fold change over IgG control from the two groups being compared is reported in the controlLogFoldChange column. This information can be used to filter non-specific binding. A controlLogFoldChange more than 1 can be used as a filter to discover specific interactors.

The results of the differential protein analysis can be visualized using `maVolPlot` function. It plots average log2 protein intensity to log2 fold change between groups compared. This enables quick visualization (Figure 11) of significantly abundant proteins between groups. `maVolPlot` could also be used to view differential protein results in a volcano plot (Figure 12) to compare the size of the fold change to the statistical significance level.

```
contrasts <- c(DSG.FA_vs_FA = "DSG.FA - FA")
diffstats <- computeDiffStats(MSnset_Pnorm, contrasts=contrasts)
```

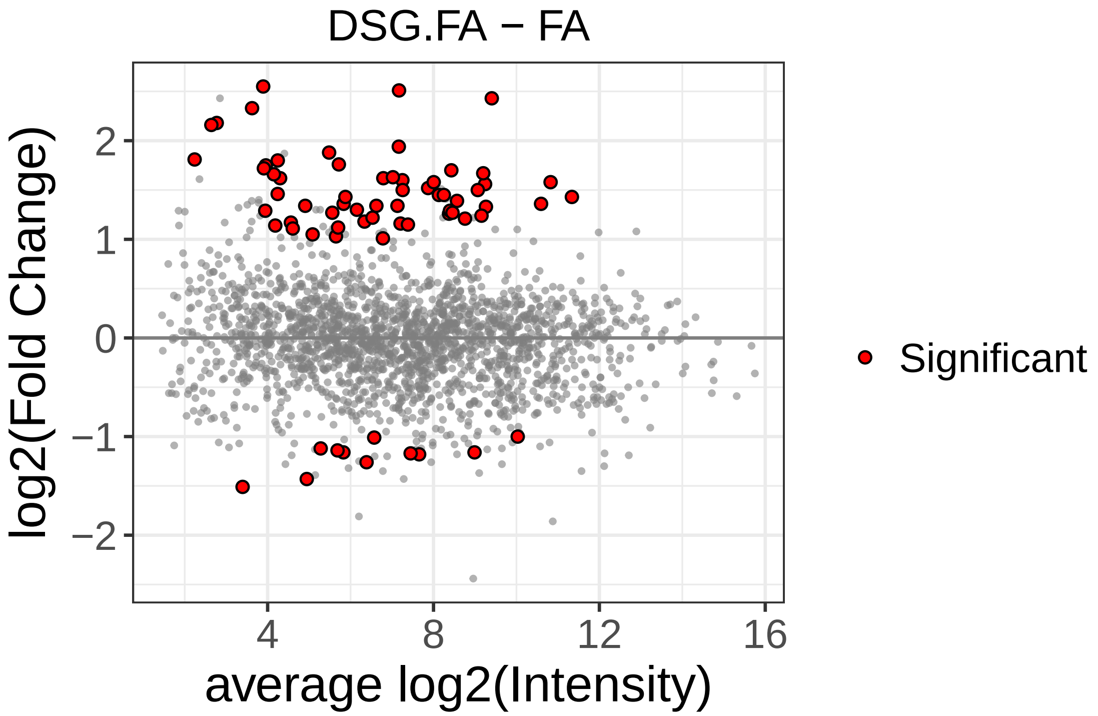

Figure 11: MA plot of the quantified proteins

```
diffexp <- getContrastResults(diffstats=diffstats, contrast=contrasts,
                              controlGroup = "IgG")
maVolPlot(diffstats, contrast = contrasts, plotType="MA", title= contrasts)
```

```
maVolPlot(diffstats, contrast = contrasts, plotType="Volcano", title= contrasts)
```

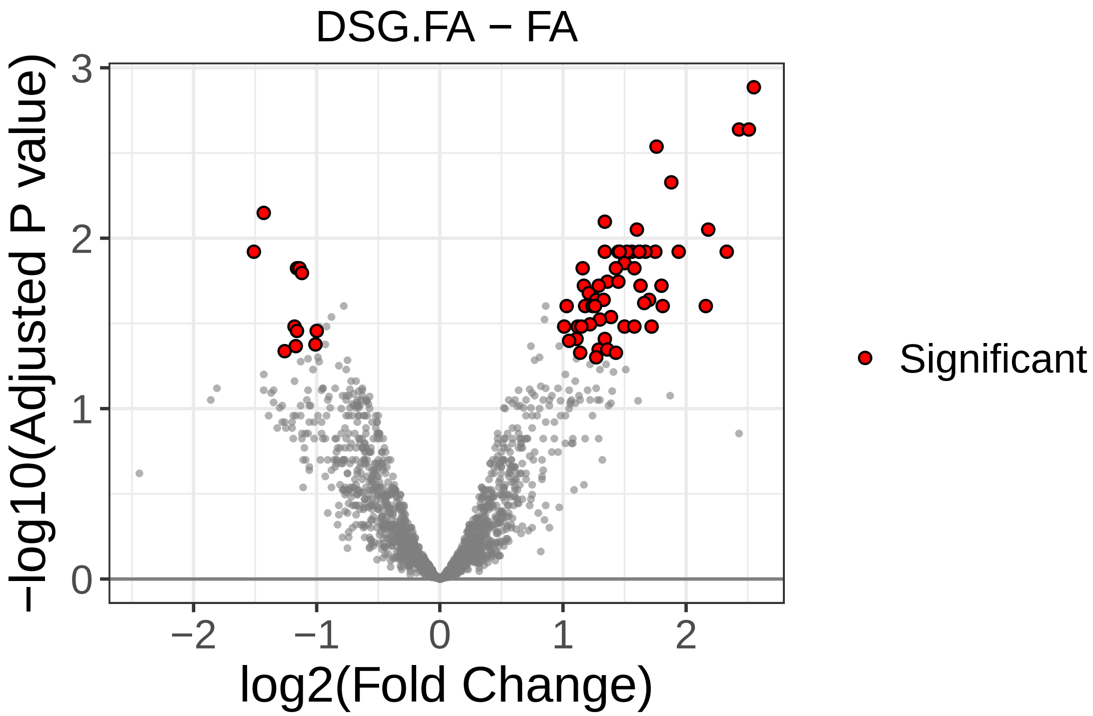

Figure 12: Volcano plot of the quantified proteins

Supplementary Note 2: qPLEXdata

(Analysis of qPLEX-RIME data and Total proteome data)

Contents

1 Description **1**

2 Usage **1**

3 Experiment 1: ER interactome in MCF7 cells **2**

4 Experiment 2: Crosslinking comparison **2**

5 Experiment 3: ER complex upon OHT treatment **3**

6 Experiment 4: Total proteome upon OHT treatment **7**

7 Experiment 5: ER interactome in PDX tumours **8**

8 Experiment 6: ER interactome in human breast cancer tumours **9**

9 Experiment 7: NCOA3 interactome in MCF7 cells **10**

10 Experiment 8: CBP interactome in MCF7 cells **10**

11 Experiment 9: POLR2A (RNA polymerase II) interactome in MCF7 cells **11**

1 Description

The report outlines the steps for the statistical analysis of the datasets generated from the application of the qPLEX-RIME approach in breast cancer cells and clinical tumour material (PDX and human breast cancer tumours). In addition, it also contains statistical analysis of total proteome data in breast cancer cells.

2 Usage

```
suppressWarnings(library(qPLEXdata))
library(dplyr)
suppressWarnings(library(qPLEXanalyzer))
data(human_anno)
```

3 Experiment 1: ER interactome in MCF7 cells

In this experiment we have used the qPLEX-RIME approach to identify ER-associated proteins. We performed replicate ER RIME pull-downs in five independent biological replicates and an equal number of matched IgG mock samples was included. As IgG samples represent the low background intensity, their intensity distribution profile is different from ER samples. Hence, normalizing the two together would have resulted in over-correction of the IgG intensity resulting in inaccurate computation of differences between the two groups. Therefore, the peptide intensities were normalized by median scaling within each group separately. The normalized peptide intensities were aggregated (by summing) to protein intensities. Thereafter, differential protein expression was performed using *limma* based analysis.

```
## load data
data(exp1_specificity)

## create MSnSet object
MSnset_data <- convertToMSnset(exp1_specificity$intensities,
                               metadata=exp1_specificity$metadata,
                               indExpData=c(6:15),Sequences=1,Accessions=5)

## Normalization
MSnset_norm <- groupScaling(MSnset_data, median)

## Summation of peptide to protein intensity
MSnset_Pnorm <- summarizeIntensities(MSnset_norm, sum, human_anno)

## Differential analysis
contrasts <- c(ER_vs_IgG = "ER - IgG")
diffstats <- computeDiffStats(MSnSetObj=MSnset_Pnorm, contrasts=contrasts)
diffexp <- getContrastResults(diffstats=diffstats, contrast=contrasts)
diffexp <- diffexp[which(diffexp$adj.P.Val < 0.01 & diffexp$log2FC >1),]
```

4 Experiment 2: Crosslinking comparison

An ER qPLEX-RIME experiment was performed to compare two different ways of cell crosslinking. MCF7 cells were crosslinked with DSG/formaldehyde (double) or with formaldehyde alone (single). Four biological replicates were obtained for each condition along with two IgG samples pooled from replicates of each group. One of the outlier sample was removed from the analysis. The peptide intensities were normalized using median scaling within the group, treating ER pull downs as one group and IgG pull downs as another. The normalized peptide intensities were aggregated (by summing) to protein intensities. Thereafter, differential protein expression was performed using *limma* based analysis.

```
## load data
data(exp2_Xlink)

## create MSnSet object
MSnset_data <- convertToMSnset(exp2_Xlink$intensities,
                               metadata=exp2_Xlink$metadata,
                               indExpData=c(7:16),Sequences=2,Accessions=6)
exprs(MSnset_data) <- exprs(MSnset_data)+0.01
```

```
MSnset_data <- MSnset_data[,-5]

## Normalization
MSnset_norm <- groupScaling(MSnset_data, median)

## Summation of peptide to protein intensity
MSnset_Pnorm <- summarizeIntensities(MSnset_norm, sum, human_anno)

## Differential analysis
contrasts <- c(DSG.FA_vs_FA = "DSG.FA - FA")
diffstats <- computeDiffStats(MSnSetObj=MSnset_Pnorm, contrasts=contrasts)
diffexp <- getContrastResults(diffstats=diffstats, contrast=contrasts,
                              controlGroup = "IgG")
diffexp <- diffexp[which(diffexp$adj.P.Val < 0.05 & abs(diffexp$log2FC) > 0.5),]
```

5 Experiment 3: ER complex upon OHT treatment

Three ER qPLEX-RIME experiments were performed to investigate the dynamics of the ER complex assembly upon 4-hydroxytamoxifen (OHT) treatment at 2h, 6h and 24h or at 24h post-treatment with the vehicle alone (ethanol). Two biological replicates of each condition were included in each experiment to finally consider a total of six replicates per time point. Additionally, MCF7 cells were treated with OHT or ethanol and cross-linked at 24h post-treatment in each experiment to be used for mock IgG pull-downs and to enable discrimination of non-specific binding.

The peptide intensities in each 10plex experiment were aggregated (by summing) to protein intensities. Thereafter, protein intensity in each sample was divided by its average intensity across all the samples and log2 transformed. The scaled protein intensities from each 10plex experiment were then combined and only proteins identified in all the experiments were kept for further analysis. To filter non-specific proteins, a *limma* based differential analysis was performed comparing ER and IgG pull-downs. This step filtered out non-specific binding. Further, a subset of dataset was created excluding IgG pull-down samples.

A linear regression method was applied on proteins (of this dataset) that were identified across all three replicate experiments to correct for the ER dependency. Finally, *limma* based statistical was applied on the regressed intensities for the identification of differentially bound proteins.

```
## load data
data(exp3_OHT_ESR1)

## create MSnSet object
MSnset_data1 <- convertToMSnset(exp3_OHT_ESR1$intensities_qPLEX1,
                                metadata=exp3_OHT_ESR1$metadata_qPLEX1,
                                indExpData=c(7:16),Sequences=2,Accessions=6)
pData(MSnset_data1)$Run <- 1
MSnset_data2 <- convertToMSnset(exp3_OHT_ESR1$intensities_qPLEX2,
                                metadata=exp3_OHT_ESR1$metadata_qPLEX2,
                                indExpData=c(7:16),Sequences=2,Accessions=6)
pData(MSnset_data2)$Run <- 2
MSnset_data3 <- convertToMSnset(exp3_OHT_ESR1$intensities_qPLEX3,
                                metadata=exp3_OHT_ESR1$metadata_qPLEX3,
```

```r
                                indExpData=c(7:16),Sequences=2,Accessions=6)
pData(MSnset_data3)$Run <- 3

## Summation of peptide to protein intensity
MSnset_P1 <- summarizeIntensities(MSnset_data1, sum, human_anno)
MSnset_P2 <- summarizeIntensities(MSnset_data2, sum, human_anno)
MSnset_P3 <- summarizeIntensities(MSnset_data3, sum, human_anno)

## Normalization
MSnset_P1 <- rowScaling(MSnset_P1,mean)
MSnset_P2 <- rowScaling(MSnset_P2,mean)
MSnset_P3 <- rowScaling(MSnset_P3,mean)

###### Compute common unique peptides
features1 <- fData(MSnset_data1)
features1 <- as.data.frame(features1[, c("Sequences",
                                       "Accessions")],
                           stringsAsFactors = F)
features2 <- fData(MSnset_data2)
features2 <- as.data.frame(features2[, c("Sequences",
                                         "Accessions")],
                           stringsAsFactors = F)
features3 <- fData(MSnset_data3)
features3 <- as.data.frame(features3[, c("Sequences",
                                         "Accessions")],
                           stringsAsFactors = F)
features <- rbind(features1,features2,features3)
features <- unique(features)
features$Sequences <- as.character(features$Sequences)
features$Accessions <- as.character(features$Accessions)
counts <- features %>% count(Accessions) %>%
  rename(Protein = Accessions, Count = n)

##### create combine MSnSet object

MSnset_P1 <- updateFvarLabels(MSnset_P1)
MSnset_P2 <- updateFvarLabels(MSnset_P2)
MSnset_P3 <- updateFvarLabels(MSnset_P3)

MSnset_P1 <- updateSampleNames(MSnset_P1)
MSnset_P2 <- updateSampleNames(MSnset_P2)
MSnset_P3 <- updateSampleNames(MSnset_P3)

suppressWarnings(MSnset_comb <- combine(MSnset_P1, MSnset_P2, MSnset_P3))
tokeep <- which(complete.cases(fData(MSnset_comb))==TRUE)
MSnset_comb <- MSnset_comb[tokeep,]
sampleNames(MSnset_comb) <- pData(MSnset_comb)$SampleName

pData(MSnset_comb)$BioRep <- c(rep(1,4),rep(2,4),c(1,2),rep(3,4),rep(4,4),c(3,4),
```

```
                           rep(5,4),rep(6,4),c(5,6))
fData(MSnset_comb) <- fData(MSnset_comb)[,c(1:4)]
colnames(fData(MSnset_comb)) <- c("Protein","Gene","Description",
                                                "GeneSymbol")
ind <- match(fData(MSnset_comb)$Protein,counts$Protein)
fData(MSnset_comb)$Count <- counts$Count[ind]

### create separate MSnSet for IgG comparision
pheno <- pData(MSnset_comb)
pheno$SampleGroup <- c(rep(c(rep("Exp",8),rep("IgG",2)),3))
pheno$SampleGroup <- factor(pheno$SampleGroup)
MSnset_IgG <- MSnset_comb
pData(MSnset_IgG) <- pheno

### Differential analysis to find ER specific interactors
contrasts <- c(
  Exp_vs_IgG = "Exp - IgG"
)

diffstats <- computeDiffStats(MSnSetObj=MSnset_IgG, contrasts=contrasts,
                              transform = FALSE)
results <- getContrastResults(diffstats=diffstats, contrast=contrasts,
                              transform = FALSE)

### create subset of protein filtering non-specific IgG
ind <- which(results$adj.P.Val < 0.01 & results$log2FC > 1)
diff_IgG <- results[ind,]
ind <- match(diff_IgG$Protein,fData(MSnset_comb)$Protein)
MSnset_subset <- MSnset_comb[ind]
IgG_ind <- which(pData(MSnset_subset)$SampleGroup == "IgG")

### perform regression analysis on dataset
MSnset_reg <- regressIntensity(MSnset_subset, controlInd=IgG_ind, ProteinId="P03372")
```

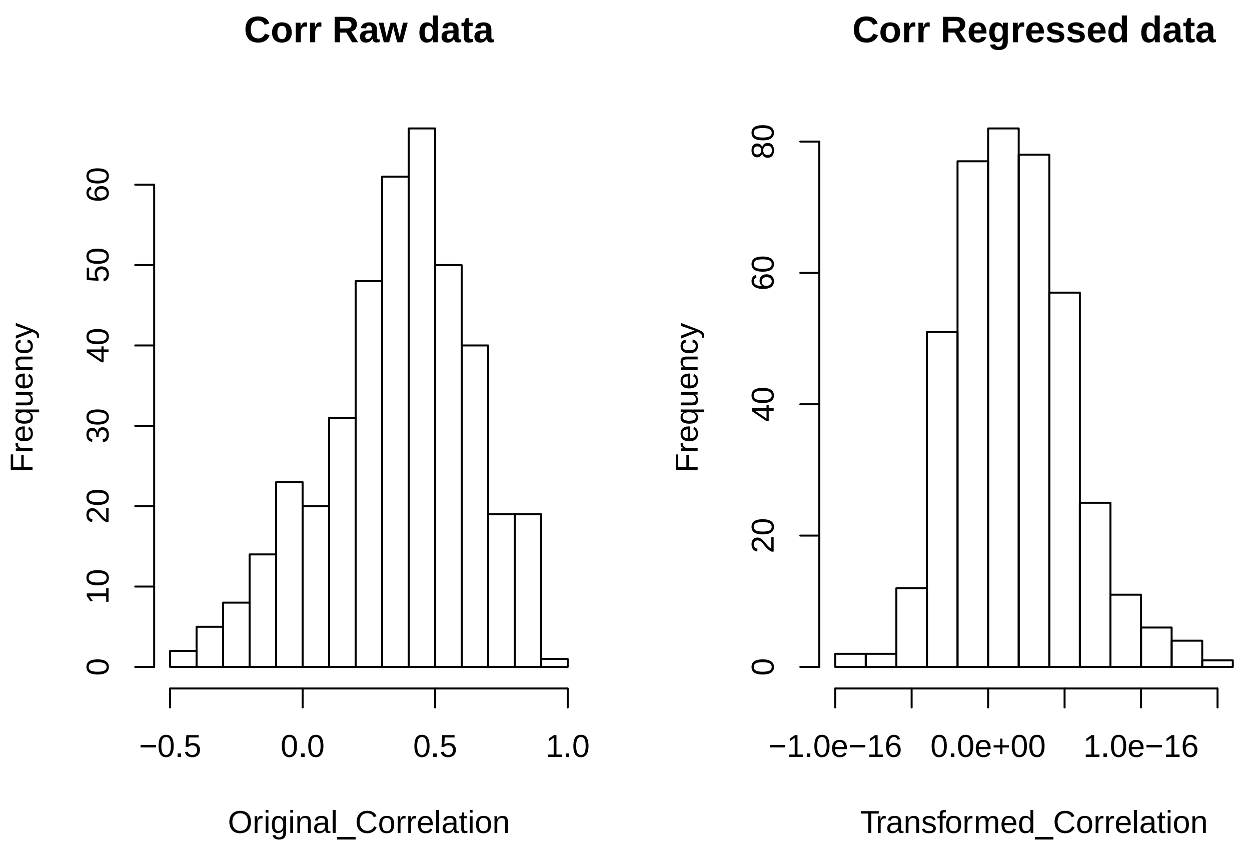


```
### Differential analysis
contrasts <- c(
  tam.2h_vs_vehicle = "tam.2h - vehicle",
  tam.6h_vs_vehicle = "tam.6h - vehicle",
  tam.24h_vs_vehicle = "tam.24h - vehicle"
)

suppressWarnings(diffstats <- computeDiffStats(MSnSetObj=MSnset_reg, contrasts=contrasts,
                              transform = FALSE))

diffexp1 <- getContrastResults(diffstats=diffstats, contrast=contrasts[1],
                              transform = FALSE)
diffexp1 <- diffexp1[which(diffexp1$adj.P.Val < 0.05 & abs(diffexp1$log2FC) > 0.5),]
diffexp2 <- getContrastResults(diffstats=diffstats, contrast=contrasts[2],
                              transform = FALSE)
diffexp2 <- diffexp2[which(diffexp2$adj.P.Val < 0.05 & abs(diffexp2$log2FC) > 0.5),]
diffexp3 <- getContrastResults(diffstats=diffstats, contrast=contrasts[3],
                              transform = FALSE)
diffexp3 <- diffexp3[which(diffexp3$adj.P.Val < 0.05 & abs(diffexp3$log2FC) > 0.5),]
```

6 Experiment 4: Total proteome upon OHT treatment

We performed two 10plex-TMT time-course experiments to study the effect of OHT on total protein levels. MCF7 cells were treated with OHT for 2h, 6h, 24h or for 24h with the vehicle alone (ethanol) and a total number of four biological replicates was obtained. The peptide intensities in each 10plex experiment were aggregated (by summing) to protein intensities and only proteins that were identified in both experiments were used for further analysis. The protein intensities were then normalized using median scaling to account for sample loading variability. Thereafter, differential protein expression was performed using *limma* based analysis.

```
## load data
data(exp4_OHT_FP)

## create MSnSet object
MSnset_data1 <- convertToMSnset(exp4_OHT_FP$FP_1,
                                metadata=exp4_OHT_FP$metadata_FP1,
                                indExpData=c(7:14),Sequences=2,Accessions=6)
pData(MSnset_data1)$Run <- 1
MSnset_data2 <- convertToMSnset(exp4_OHT_FP$FP_2,
                                metadata=exp4_OHT_FP$metadata_FP2,
                                indExpData=c(7:14),Sequences=2,Accessions=6)
pData(MSnset_data2)$Run <- 2

## Summation of peptide to protein intensity
MSnset_P1 <- summarizeIntensities(MSnset_data1, sum, human_anno)
MSnset_P2 <- summarizeIntensities(MSnset_data2, sum, human_anno)


### Computing common unique peptides
features1 <- fData(MSnset_data1)
features1 <- as.data.frame(features1[, c("Sequences","Accessions")],
                           stringsAsFactors = F)
features2 <- fData(MSnset_data2)
features2 <- as.data.frame(features2[, c("Sequences","Accessions")],
                           stringsAsFactors = F)
features <- rbind(features1,features2)
features <- unique(features)
features$Sequences <- as.character(features$Sequences)
features$Accessions <- as.character(features$Accessions)
counts <- features %>% count(Accessions) %>%
  rename(Protein = Accessions, Count = n)

##### create combine MSnSet object

MSnset_P1 <- updateFvarLabels(MSnset_P1)
MSnset_P2 <- updateFvarLabels(MSnset_P2)
MSnset_P1 <- updateSampleNames(MSnset_P1)
MSnset_P2 <- updateSampleNames(MSnset_P2)

suppressWarnings(MSnset_comb <- combine(MSnset_P1, MSnset_P2))
```

```
tokeep <- which(complete.cases(fData(MSnset_comb))==TRUE)
MSnset_comb <- MSnset_comb[tokeep,]
sampleNames(MSnset_comb) <- pData(MSnset_comb)$SampleName
fData(MSnset_comb) <- fData(MSnset_comb)[,c(1:4)]
colnames(fData(MSnset_comb)) <- c("Protein","Gene","Description",
                                  "GeneSymbol")
ind <- match(fData(MSnset_comb)$Protein,counts$Protein)
fData(MSnset_comb)$Count <- counts$Count[ind]

## Normalization
MSnset_Pnorm <- normalizeScaling(MSnset_comb, median)

## Differential analysis
contrasts <- c(
  tam.2h_vs_vehicle = "tam.2h - vehicle",
  tam.6h_vs_vehicle = "tam.6h - vehicle",
  tam.24h_vs_vehicle = "tam.24h - vehicle"
)
batchEffect <- c("Run", "BioRep")

diffstats <- computeDiffStats(MSnset_Pnorm, contrasts=contrasts,
                              batchEffect=batchEffect)

diffexp1 <- getContrastResults(diffstats=diffstats, contrast=contrasts[1])
diffexp1 <- diffexp1[which(diffexp1$adj.P.Val < 0.05 & abs(diffexp1$log2FC) > 0.5),]
diffexp2 <- getContrastResults(diffstats=diffstats, contrast=contrasts[2])
diffexp2 <- diffexp2[which(diffexp2$adj.P.Val < 0.05 & abs(diffexp2$log2FC) > 0.5),]
diffexp3 <- getContrastResults(diffstats=diffstats, contrast=contrasts[3])
diffexp3 <- diffexp3[which(diffexp3$adj.P.Val < 0.05 & abs(diffexp3$log2FC) > 0.5),]
```

7 Experiment 5: ER interactome in PDX tumours

An ER qPLEX-RIME experiment was performed using three independent ER+ human Patient Derived Xenograft (PDX) tumours. Cryosections of each tumour were double-crosslinked and each tumour was split in two parts that were used for ER and IgG RIME pull-down assays. One of the tumours was split in three different parts to be used as ER or IgG qPLEX-RIME in order to assess technical variability. The peptide intensities were normalized by median scaling within each group separately. The normalized peptide intensities were aggregated (by summing) to protein intensities. Thereafter, differential protein expression was performed using *limma* based analysis.

```
## load data
data(exp5_PDX)

## create MSnSet object
MSnset_data <- convertToMSnset(exp5_PDX$intensities, metadata=exp5_PDX$metadata,
                               indExpData=c(7:16), Sequences=2,Accessions=6)

## Exclude outlier and techical replicate samples
```

```r
MSnset_data <- MSnset_data[,-c(7:10)]

## Normalization
MSnset_norm <- groupScaling(MSnset_data, median)

## Summation of peptide to protein intensity
MSnset_Pnorm <- summarizeIntensities(MSnset_norm, sum, human_anno)

## Differential analysis
contrasts <- c(PDX_vs_IgG = "PDX - IgG")

diffstats <- computeDiffStats(MSnset_Pnorm, contrasts=contrasts)
diffexp <- getContrastResults(diffstats=diffstats, contrast=contrasts)
diffexp <- diffexp[which(diffexp$adj.P.Val < 0.05 & diffexp$log2FC > 1),]
```

8 Experiment 6: ER interactome in human breast cancer tumours

An ER qPLEX-RIME experiment was performed using five independent ER-positive human breast cancer tumours. Cryosections of each tumour were double-crosslinked and each tumour was split in two parts that were used for ER and IgG RIME pull-down assays. The peptide intensities were normalized by median scaling within each group separately. The normalized peptide intensities were aggregated (by summing) to protein intensities followed by differential protein expression using *limma* based analysis.

```r
## load data
data(exp6_ER)

## create MSnSet object
MSnset_data <- convertToMSnset(exp6_ER$intensities, metadata=exp6_ER$metadata,
                               indExpData=c(6:15), Sequences=2, Accessions=5,
                               rmMissing=FALSE)
exprs(MSnset_data)[is.na(exprs(MSnset_data))] <- 0
exprs(MSnset_data) <- exprs(MSnset_data)+0.01

## Normalization
MSnset_norm <- groupScaling(MSnset_data, median, Grp="SampleGroup")

## Summation of peptide to protein intensity
MSnset_Pnorm <- summarizeIntensities(MSnset_norm, sum, human_anno)

## Differential analysis
contrasts <- c(ER_vs_IgG = "ER - IgG")
diffstats <- computeDiffStats(MSnset_Pnorm, contrasts=contrasts)
diffexp <- getContrastResults(diffstats=diffstats, contrast=contrasts)
diffexp <- diffexp[which(diffexp$adj.P.Val < 0.01 & diffexp$log2FC >1),]
```

9 Experiment 7: NCOA3 interactome in MCF7 cells

In this experiment we have used the qPLEX-RIME method to identify and characterize NCOA3 (SRC-3) associated proteins. We performed NCOA3 RIME pull-downs in five independent biological replicates and in five matched IgG mock samples. The peptide intensities were normalized by median scaling within each group separately. The normalized peptide intensities were aggregated (by summing) to protein intensities followed by differential protein expression using *limma* based analysis.

```
## load data
data(exp7_NCOA3)

## create MSnSet object
MSnset_data <- convertToMSnset(exp7_NCOA3$intensities, metadata=exp7_NCOA3$metadata,
                               indExpData=c(7:16), Sequences=2, Accessions=6,
                               rmMissing=FALSE)
exprs(MSnset_data)[is.na(exprs(MSnset_data))] <- 0
exprs(MSnset_data) <- exprs(MSnset_data)+0.01

## Normalization
MSnset_norm <- groupScaling(MSnset_data, median, Grp="SampleGroup")

## Summation of peptide to protein intensity
MSnset_Pnorm <- summarizeIntensities(MSnset_norm, sum, human_anno)

## Differential analysis
contrasts <- c(NCOA3_vs_IgG = "NCOA3 - IgG")
diffstats <- computeDiffStats(MSnset_Pnorm, contrasts=contrasts)
diffexp <- getContrastResults(diffstats=diffstats, contrast=contrasts)
diffexp <- diffexp[which(diffexp$adj.P.Val < 0.01 & diffexp$log2FC >1),]
```

10 Experiment 8: CBP interactome in MCF7 cells

A qPLEX-RIME experiment was designed for the characterization of the CBP (CREB-binding protein) interactome. Five independent biological replicates of CBP RIME pull-downs and five IgG RIME pull-downs were prepared for this experiment. The peptide intensities were normalized by median scaling within each group separately. The normalized peptide intensities were aggregated (by summing) to protein intensities followed by differential protein expression using *limma* based analysis.

```
## load data
data(exp8_CBP)

## create MSnSet object
MSnset_data <- convertToMSnset(exp8_CBP$intensities, metadata=exp8_CBP$metadata,
                               indExpData=c(7:16), Sequences=2, Accessions=6,
                               rmMissing=FALSE)
exprs(MSnset_data)[is.na(exprs(MSnset_data))] <- 0
exprs(MSnset_data) <- exprs(MSnset_data)+0.01

## Normalization
```

```
MSnset_norm <- groupScaling(MSnset_data, median, Grp="SampleGroup")

## Summation of peptide to protein intensity
MSnset_Pnorm <- summarizeIntensities(MSnset_norm, sum, human_anno)

## Differential analysis
contrasts <- c(CREBBP_vs_IgG = "CREBBP - IgG")
diffstats <- computeDiffStats(MSnset_Pnorm, contrasts=contrasts)
diffexp <- getContrastResults(diffstats=diffstats, contrast=contrasts)
diffexp <- diffexp[which(diffexp$adj.P.Val < 0.01 & diffexp$log2FC >1),]
```

11 Experiment 9: POLR2A (RNA polymerase II) interactome in MCF7 cells

The qPLEX-RIME method was applied for the characterization of the largest and catalytic component of RNA polymerase II (RPB1). Particularly, the phosphorylated form at Serine 5 in the C-terminal domain (CTD) was used as the bait protein. Five biological replicates of RNA polymerase II RIME pull-downs and five IgG pull-downs were included for the identification and characterization of RNA polymerase II-associated proteins. The peptide intensities were normalized by median scaling within each group separately. The normalized peptide intensities were aggregated (by summing) to protein intensities followed by differential protein expression using *limma* based analysis.

```
## load data
data(exp9_PolII)

## create MSnSet object
MSnset_data <- convertToMSnset(exp9_PolII$intensities, metadata=exp9_PolII$metadata,
                               indExpData=c(7:16), Sequences=2, Accessions=6,
                               rmMissing=FALSE)
exprs(MSnset_data)[is.na(exprs(MSnset_data))] <- 0
exprs(MSnset_data) <- exprs(MSnset_data)+0.01

## Normalization
MSnset_norm <- groupScaling(MSnset_data, median, Grp="SampleGroup")

## Summation of peptide to protein intensity
MSnset_Pnorm <- summarizeIntensities(MSnset_norm, sum, human_anno)

## Differential analysis
contrasts <- c(POLR2A_vs_IgG = "POLR2A - IgG")
diffstats <- computeDiffStats(MSnset_Pnorm, contrasts=contrasts)
diffexp <- getContrastResults(diffstats=diffstats, contrast=contrasts)
diffexp <- diffexp[which(diffexp$adj.P.Val < 0.01 & diffexp$log2FC >1),]
```
