# Supplementary material for: A quantitative mass spectrometry-based approach to monitor the dynamics of endogenous chromatin-associated protein complexes
Source: Nat Commun. 2018 Jun 13;9:2311. doi: 10.1038/s41467-018-04619-5 (PMC5998130; doi:10.1038/s41467-018-04619-5)
Supplement: Supplementary file 14 — Description of Additional Supplementary files [file 41467_2018_4619_MOESM14_ESM.pdf]

Description of Additional Supplementary files

File Name: Supplementary Data Set 1.
Description: Result table for the duplicate non-quantitative ERα and IgG RIME experiments to identify ERα-associated interactors at peptide FDR<1%. The presence or absence of a particular protein in each sample is shown in the binary matrix as 1 or 0 respectively. As specific ERα interactors we consider proteins with presence in both ERα RIME samples and absence in both IgG RIME samples.

File Name: Supplementary Data Set 2.
Description: The qPLEXanalyzer output results from an ERα qPLEX-RIME experiment, where we included five ERα qPLEX-RIME pull-downs in five independent biological replicates with the respective number of matched IgG control samples. Samples were single crosslinked with formaldehyde and the qPLEX-RIME raw data processing quantified 2,955 proteins across all samples at FDR<1%. For the calculation of log2FC, the ERα samples were compared to IgG controls.

File Name: Supplementary Data Set 3.
Description: The qPLEXanalyzer output obtained from an ERα qPLEX-RIME experiment that was performed to compare two different ways of cell crosslinking. MCF7 cells were double crosslinked with DSG/formaldehyde (double) or with formaldehyde (FA) alone (single). Biological replicates were obtained for each condition with the respective IgG pooled samples from each replicate. All samples were compared to IgG controls for the discrimination of unspecific binding (controlLog2FC). Double crosslinked samples were compared with the single crosslinked ones for the identification of proteins with higher or lower enrichment after the double crosslinking (log2FC).

File Name: Supplementary Data Set 4.
Description: The qPLEXanalyzer output results from a CBP (CREB-binding protein) qPLEX-RIME experiment, where we included five CBP qPLEX-RIME pull- downs in five independent biological replicates with the respective number of matched IgG control samples. Samples were double crosslinked using DSG and formaldehyde and the qPLEX-RIME raw data processing quantified 1,437 proteins across all samples at FDR<1%. For the calculation of log2FC the CBP samples were compared to IgG controls.

File Name: Supplementary Data Set 5.
Description: The qPLEXanalyzer output contains the results from a NCOA3 (SRC-3) qPLEX-RIME experiment. In this experiment, five biological replicates of NCOA3 qPLEX-RIME pull-downs with the respective number of matched IgG control samples were included. Samples were double crosslinked and 1,135 proteins were quantified across all samples (FDR<1%). For the calculation of log2FC the NCOA3 samples were compared to IgG controls.

File Name: Supplementary Data Set 6.
Description: The qPLEXanalyzer output results from a phospho-RNA

polymerase II (POLR2A) qPLEX-RIME experiment, where we included five POLR2A independent biological replicates and an equal number of matched IgG controls. Samples were double crosslinked and the qPLEX-RIME raw data processing quantified 1,442 proteins across all samples at FDR<1%. For the calculation of log2FC the POLR2A samples were compared to IgG controls.

File Name: Supplementary Data Set 7.
Description: The results from the RNA-seq analysis of MCF7 cells treated with 100nM OHT for 2h, 6h and 24h or 24h with vehicle alone (ethanol). Six biological replicates of each time point were included in the analysis. We have identified 27,520 genes of which 1,187 were significantly regulated in at least one time point compared to the vehicle.

File Name: Supplementary Data Set 8.
Description: The qPLEXanalyzer output results from three ERα qPLEX-RIME experiments (3×10plex) using independently prepared biological replicates. MCF7 cells were treated with 100nM OHT for 2h, 6h and 24h or after 24h treatment with the vehicle alone (ethanol). Two biological replicates of each condition were included in each experiment, resulting in a total of six replicates per time point. This table contains only the ERα specific proteins filtered based on IgG pull-downs included in the same experiment. The quantification values represent the log2 row-mean scaled intensities after regression correction.

File Name: Supplementary Data Set 9.
Description: The qPLEXanalyzer output of two 10plex-TMT time-course experiments that were performed to study the effect of OHT on total protein levels. MCF7 cells were treated with OHT for 2h, 6h and 24h or for 24h with the vehicle alone (ethanol) and a total number of four biological replicates were obtained. This table contains 7,943 proteins quantified in both TMT experiments.

File Name: Supplementary Data Set 10.
Description: The qPLEXanalyzer output results from an ERα qPLEX-RIME experiment using three independent ER+ human Patient Derived Xenograft tumours. Each tumour was split in two parts, for ERα and matched IgG pull-downs. All ERα samples were compared to IgG controls for the identification of ERα-associated interactors.

File Name: Supplementary Data Set 11.
Description: The qPLEXanalyzer output results from an ERα qPLEX-RIME experiment in five independent ERα positive human breast cancer tumours. Each tumour was split in two parts, for ERα and matched IgG pull-downs. The ERα samples were compared to IgG controls for the identification of ERα-associated interactors.
